# Characterization of demographic data, clinical signs, comorbidities, and outcomes according to the race in hospitalized individuals with COVID-19 in Brazil: An observational study

**DOI:** 10.7189/jogh.12.05027

**Published:** 2022-07-25

**Authors:** Nathália MS Sansone, Matheus N Boschiero, Felipe E Valencise, Camila VC Palamim, Fernando AL Marson

**Affiliations:** 1 Laboratory of Cell and Molecular Tumor Biology and Bioactive Compounds, São Francisco University, Bragança Paulista, São Paulo, Brazil; 2 Laboratory of Human and Medical Genetics, São Francisco University, Bragança Paulista, São Paulo, Brazil

## Abstract

**Background:**

Brazil is a multiracial country with five major official races: White, Black, individuals with multiracial backgrounds, Asian, and Indigenous. Brazil is also one of the epicentres of the Coronavirus Disease (COVID)-19 pandemic. Thus, we evaluated how the races of the Brazilian population contribute to the outcomes in hospitalized individuals with COVID-19, and we also described the clinical profile of the five official Brazilian races.

**Methods:**

We performed an epidemiological analysis for the first 67 epidemiological weeks of the COVID-19 pandemic in Brazil (from February 22, 2020, to April 04, 2021) using the data available at OpenDataSUS of the Brazilian Ministry of Health, a data set containing data from Brazilian hospitalized individuals. We evaluated more than 30 characteristics, including demographic data, clinical symptoms, comorbidities, need for intensive care unit and mechanical ventilation, and outcomes.

**Results:**

In our data, 585 655 hospitalized individuals with a positive result in SARS-CoV-2 real-time chain reaction (RT-PCR) were included. Of these total, 309 646 (52.9%) identified as White, 31 872 (5.4%) identified as Black, 7108 (1.2%) identified as Asian, 235 108 (40.1%) identified as individuals with multiracial background, and 1921 (0.3%) identified as Indigenous. The multivariate analysis demonstrated that race was significative to predict the death being that Black (OR = 1.43; 95% CI = 1.39-1.48), individuals with multiracial background (OR = 1.36; 95% CI = 1.34-1.38), and Indigenous (OR = 1.91; 95% CI = 1.70-2.15) races were more prone to die compared to the White race. The Asian individuals did not have a higher chance of dying due to SARS-CoV-2 infection compared to White individuals (OR = 0.99; 95% CI = 0.94-1.06). In addition, other characteristics contributed as such as being male (OR = 1.17; 95% CI = 1.16-1.19), age (mainly, +85 years old – OR = 23.02; 95% CI = 20.05-26.42) compared to 1-year-old individuals, living in rural areas (OR = 1.22; 95% CI = 1.18-1.26) or in peri-urban places (OR = 1.25; 95% CI = 1.11-1.40), and the presence of nosocomial infection (OR = 1.91; 95% CI = 1.82-2.01). Among the clinical symptoms, the main predictors were dyspnoea (OR = 1.25; 95% CI = 1.23-1.28), respiratory discomfort (OR = 1.30; 95% CI = 1.28-1.32), oxygen saturation <95% (OR = 1.40; 95% CI = 1.38-1.43). Also, among the comorbidities, the main predictors were the presence of immunosuppressive disorder (OR = 1.44; 95% CI = 1.39-1.49), neurological disorder (OR = 1.21; 95% CI = 1.17-1.25), hepatic disorder (OR = 1.41; 95% CI = 1.34-1.50), diabetes mellitus (OR = 1.40; 95% CI = 1.37-1.42), cardiopathy (OR = 1.13; 95%CI = 1.11-1.14), hematologic disorder (OR = 1.34; 95% CI = 1.24-1.43), Down syndrome (OR = 1.61; 95% CI = 1.43-1.81), renal disease (OR = 1.15; 95% CI = 1.11-1.18), and obesity (OR = 1.18; 95% CI = 1.15-1.21). Individuals on intensive care unit (OR = 2.25; 95% CI = 2.22-2.29) and on invasive (OR = 10.92; 95% CI = 10.66-11.18) or non-invasive (OR = 1.33; 95% CI = 1.30-1.35) mechanical ventilation were more prone to die.

**Conclusions:**

Alongside several clinical symptoms and comorbidities, we associated race with an enhanced risk of death in Black individuals, individuals with multiracial backgrounds, and Indigenous peoples.

The Coronavirus Disease (COVID)-19 pandemic was one of the deadliest in the world, which led the scientific community to make a worldwide effort to identify those individuals at risk of the worst outcomes. Several clinical and epidemiologic characteristics, such as obesity, diabetes mellitus, systemic arterial hypertension, and older age were associated with increased severity and mortality [1-4]. In the same way, a growing body of evidence indicates that race also plays a vital role in the COVID-19 outcomes, such as enhanced severity or even mortality in minority groups, such as the Black race and Indigenous peoples [5-9]. In addition, the race is an essential feature due to its association with comorbidities profile (genetic and environmental interaction) and health care access in different countries which might play a role in the other case fatality rates due to severe acute respiratory syndrome coronavirus 2 (SARS-CoV-2) in each race [6,8,10-14].

A meta-analysis, which compiled nearly 50 studies, observed a higher risk of intensive care unit (ICU) in African Americans and Asian Americans than in White individuals [15]. In the same way, another meta-analysis observed increased severity in COVID-19 cases in minority groups, perhaps, due to increased comorbidities in this group [16]. Although different genetic backgrounds might help explain the various impacts of COVID-19 across different races [17-19], differences in socioeconomic and demographic traits might play a more critical role in the burden of the COVID-19 pandemic. For instance, several previous studies observed that the SARS-CoV-2 infection rates in different races are associated with low socioeconomic and income status [20-22]. Furthermore, a report showed that minority groups, especially the Black ones, have a higher prevalence of comorbidities, such as systemic arterial hypertension, diabetes mellitus, and obesity, which might also play a role in the differences in severity across races [10,23].

The socioeconomic and demographic traits enlighten the role race plays in COVID-19. In Brazil, it is no different; there is a higher impact of the pandemic on Indigenous peoples [8,12,13,24]. In the same way, other races, such as Black people or individuals with multiracial backgrounds (*Pardos*), were associated with higher mortality and worse outcomes in COVID-19 in Brazilian individuals [6,7,25,26]. However, Brazil is a different country across all the states, with high variability in socioeconomic and demographic indexes [27,28], which could lead to other risks among the races across the Brazilian states and Federal District since those indexes might influence the outcomes of COVID-19 [20]. Furthermore, the Brazilian population is extremely varied, with high variability in its genetic background [29] and races [28]. Several previous Brazilian studies focused on race and its association with the COVID-19 progression in Brazilian cities and states [25,30-33]. Most of the published studies reported a nationwide survey comprising all the Brazilian territory, despite the different socioeconomic and demographic indexes across the Brazilian states and Federal District [5,7,12,26,34,35]. National surveys regarding race and COVID-19 are essential to characterize a country as a whole and evaluate how race can impact a country-based population, despite regional differences. Besides the significant number of studies performed about the Brazilian races, no studies assessed the complete profile for demographic data, clinical symptoms, comorbidities, and outcomes regarding the five official Brazilian races.

Thus, the primary aim of this study is to evaluate how race can impact COVID-19 outcomes in a country-based population. We also aimed to describe the demographic data, clinical symptoms, and comorbidities differences between the five “official” Brazilian races (White, Black, individuals with multiracial background**,** Indigenous, and Asian) in COVID-19. In such a scenario, the article elucidates how different patterns and multifactorial junctions for all the Brazilian races interact, so those disparities are explained to make possible a real effort in public health to prioritize those races with greater case fatality rates, promoting equity.

## METHODS

In the present study, we performed an epidemiological analysis using the data available at OpenDataSUS (https://opendatasus.saude.gov.br/). The Brazilian Ministry of Health computed the data according to severe acute respiratory infection (SARI) surveillance data in the Information System Platform for Epidemiological Surveillance of Influenza (SIVEP-Flu, acronym for Sistema de *Informação de Vigilância Epidemiológica da Gripe*). The SIVEP-Flu data set has been used since 2009, and it was mainly implemented due to the H1N1 virus pandemic (Swine Flu) and is also responsible for centralizing the SARI reports for the Brazilian Ministry of Health [36]. Several previous studies also used this data set, mainly for the COVID-19 pandemic [5,12,27,34,36-43].

We performed the analysis to evaluate five topics: (i) the identification of SARS-CoV-2 patients’ characteristics for demographic data, clinical symptoms, comorbidities, and hospitalization information according to the race; (ii) to identify the risk of death due to SARS-CoV-2 infection using race and type of ventilation support as predictors; (iii) to identify the risk of death according to the SARS-CoV-2 patients’ characteristics for demographic data, clinical symptoms, comorbidities, and hospitalization information according to the race; (iv) to demonstrate the SARS-CoV-2 patients’ characteristics for demographic data, clinical symptoms, comorbidities, and hospitalization information according to the races; and (v) to perform a multivariate analysis to identify the main features associated with the death due to SARS-CoV-2 infection in Brazilian hospitalized individuals due to severe acute respiratory syndrome (SARS).

In our study, we performed the following workflow:

a) race classification: Although the meaning of race and ethnicity might overlap, the Merriam-Webster dictionary defines race as “a group sharing outward physical characteristics and some commonalities of culture and history,” whereas ethnicity is defined as “markers acquired from the group with which one share cultural, traditional, and familial bonds” [15]. Thus, from now on, the term race will be used and not ethnicity since it is most suitable for our variable. Also, the Brazilian Institute of Geography and Statistics classifies Brazilian citizens into five official races as follows: White, Black, individuals with multiracial background (*Pardos*), Asian, and Indigenous peoples [44]. The race was self-declared, and the individuals should identify themselves by selecting only one category.

b) data acquisition: we obtained the .csv file from the OpenDataSUS (https://opendatasus.saude.gov.br/). We downloaded the file and opened it using the Statistical Package for the Social Sciences (SPSS) software (IBM SPSS Statistics for Macintosh, Version 27.0, IBM inc, Armonk NY, USA). After that, we obtained the data for the first 67 epidemiological weeks of the COVID-19 pandemic in Brazil (from February 22, 2020, to April 04, 2021). Inclusion criteria: we only included the individuals who presented a SARS-CoV-2 real-time polymerase chain reaction (RT-PCR)-positive and presented the races described in the data set.

c) data set adjustments and data description: in the data set was possible to obtain the following information: (i) demographic profile, including sex (male and female), age (<1-year-old, 1 to 12 years old, 13 to 24 years old, 25 to 60 years old, 61 to 72 years old, 73 to 85 years old, and +85 years old), and place of residence (urban, rural, and peri-urban); (ii) data for viruses infection as a residence in a place with a Flu outbreak, presence of hospital-acquired infection (nosocomial), and antiviral drug use to treat Influenzae virus infection; (iii) presence of comorbidities (comorbidities (any), cardiopathy, hematologic disorder, Down syndrome, hepatic disorder, asthma, diabetes mellitus, neurological disorder, systemic arterial hypertension, chronic respiratory disorders, immunosuppressive disorder, renal disease, obesity, and others (excluding the previous ones)); (iv) clinical symptoms related to SARS (fever, cough, sore throat, dyspnoea, respiratory discomfort, oxygen saturation, diarrhoea, vomit, abdominal pain, fatigue, loss of smell, loss of taste, myalgia, and others symptoms); (v) need for ICU treatment and need for mechanical ventilation support (invasive mechanical ventilation, non-invasive mechanical ventilation, and mechanical ventilation was not required); (vi) length of hospital stay (days), length of stay in the ICU (days), and outcomes (recovered or death). We calculated the length of hospital stays and length of stay in the ICU using the SPSS software by the differences between the date of hospitalization and the date of hospital discharge or death and the differences between the date of hospitalization in the ICU and the discharge from ICU due to death or recovery, respectively. For accuracy, three authors (FALM, FEV, and MNB) revised the epidemiological data from the individuals with SARS-CoV-2 infection obtained in the data set. We also coded the categorical data using numbers to perform the attribute of missing data and descriptive and inferential statistical analyses. We saved the SPSS data set as a .xls file to perform the imputation for the missing data.

d) Missing data analysis: we performed the inclusion of missing data for some features because (i) we had more than 5% of missingness, (ii) we did not have missing data only for the dependent variable, and (iii) we assumed that the variables were missing completely at random. Also, we excluded three characteristics that had more than 40% of missingness which included the image exams (X-Ray results and high-resolution computed tomography of the chest), educational level, and post-partum. We imputed the missing data using the XLSTAT Statistical Software for Excel (Addinsoft Inc, Paris, Île-de-France, France). For the qualitative (categorical data), we estimated the missing data using the NIPALS (Nonlinear Iterative Partial Least Squares) algorithm; also, for the quantitative (numerical data), we estimated the missing data using the MCMC (Markov chain Monte Carlo) multiple imputation algorithm. The XLSTAT Statistical Software generated a new Excel data set used to perform the statistical analyses in the SPSS software.

e) Descriptive analysis: we did the descriptive analyses using the number of individuals (N) and the percentage (%) for categorical data. We also presented the mean ± standard deviation [or mean and 95% confidence interval (95% CI)] for the numerical data. For the inferential statistical analyses, when possible, we also presented the odds ratio (OR) and 95% confidence interval (95% CI) (see below).

f) Bivariate analysis: We performed the Bivariate statistical analysis using the SPSS software and OpenEpi software (OpenEpi: Open-Source Epidemiologic Statistics for Public Health, Version. www.OpenEpi.com, 2013/04/06). We used the χ^2^ (and Fisher Exact test once) to evaluate the frequency of death according to the epidemiological data among the individuals with positive SARS-CoV-2 infection. We calculated the OR and 95% CI to estimate the association of each marker with the presence of death. We calculated the OR using the OpenEpi software for 2 × 2 tables, including the value for each patient characteristic. We summarized the results in tables and figures. We built the figures using GraphPad Prism version 8.0.0 for Mac (http://www.graphpad.com, GraphPad Software, San Diego CA, USA).

g) Multivariate analysis: We did the multivariate analysis using the Binary Logistic Regression Model with the Backward Stepwise method. We included in the regression model the markers with *P* ≤ 0.05 in the bivariate analysis. The response variable was the health outcome (recovery or death). We included 33 patients’ characteristics in the multivariate analysis. We did not use the data for comorbidities (any) or other, symptoms (other), and the patients’ features with a *P* > 0.05. We presented in the Logistic Regression Model the (i) B coefficient (including the SE (standard error)), which for the constant was called intercept; (ii) the Wald χ^2^ test and its significance; (iii) degrees of freedom (df) for the Wald χ^2^ test; and (iv) Exp(B) which represents the exponentiation of the B coefficient (OR) including its 95% CI. Before performing the statistical analysis, we tested the markers for multicollinearity considering cut-offs >0.1 for tolerance and >10 for variance inflation factor.

The study does not require approval from the ethics committee, as the data were publicly available, not containing personal information about the individuals, thus being exempted from ethical opinion.

## RESULTS

### Distribution of the Brazilian individuals with SARS due to SARS-CoV-2 infection in Brazil

**Figure 1:**
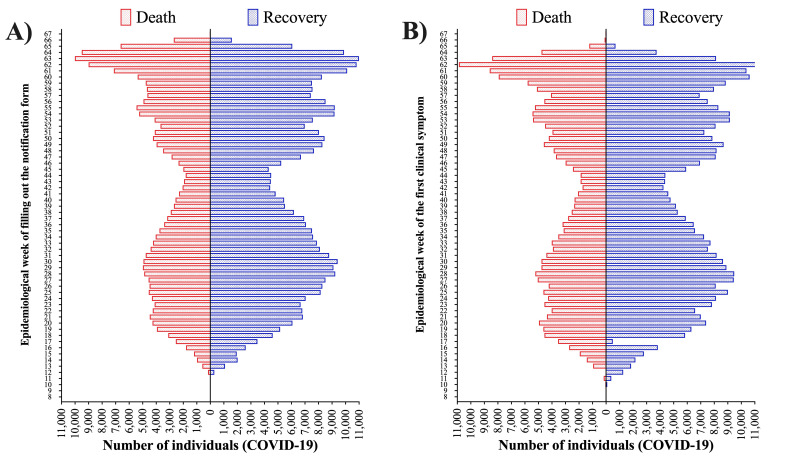
Hospitalization due to severe acute respiratory syndrome (SARS) individuals due to Coronavirus Disease (COVID)-19 in Brazil. A) Number of hospitalized SARS individuals due to COVID-19 according to the epidemiologic week of filling out the notification form and the outcomes. B) Number of hospitalized SARS individuals due to COVID-19 according to the epidemiologic week of the first symptoms and the outcomes. We retrieved the individuals’ data from the Brazilian Ministry of Health (https://opendatasus.saude.gov.br/) platform and corresponded to one year of the pandemic (from February 22, 2020, to April 04, 2021).

**Figure 2:**
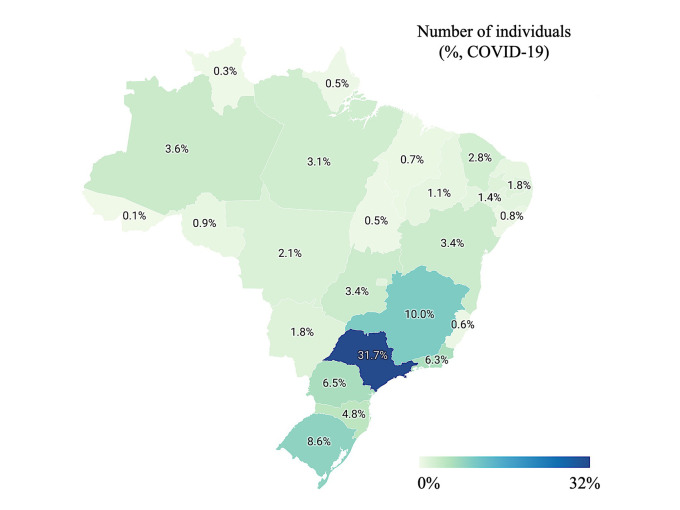
Distribution of the individuals with Coronavirus Disease (COVID)-19 according to the Brazilian States and Federal district. We presented the data as a percentage. We retrieved the individuals’ data from the Brazilian Ministry of Health (https://opendatasus.saude.gov.br/) platform and corresponded to one year of the pandemic (from February 22, 2020, to April 04, 2021).

We included 585 655 individuals with COVID-19, 309 646 (52.9%) identified as White, 31 872 (5.4%) identified as Black, 7108 (1.2%) identified as Asian, 235 108 (40.1%) identified as individuals with multiracial background, and 1921 (0.3%) identified as Indigenous (**Table 1**). We presented the individuals’ distribution according to the epidemiological week of the notification of SARS cases due to SARS-CoV-2 infection (COVID-19) in Table S1 in the **Online Supplementary Document** and **Figure 1** (Panel A) and the epidemiological week of the first clinical symptom in Table S2 in the **Online Supplementary Document** and **Figure 1** (Panel B). Also, we presented the individuals’ distribution (percentage) according to the Brazilian territory (states and Federal District) in Table S3 in the **Online Supplementary Document** and **Figure 2**.

**Table 1:** Demographic, clinical symptoms, comorbidities, and hospitalization information of individuals with the severe acute respiratory syndrome (SARS) due to SARS-CoV2 infection in Brazil*

Data	Category	N (%)
Sex	Female	261 445 (44.6%)
	Male	324 210 (55.4%)
Age – years old	<1	2833 (0.5%)
	1 to 12	5376 (0.9%)
	13 to 24	10 647 (1.8%)
	25 to 60	261 523 (44.7%)
	61 to 72	148 509 (25.4%)
	73 to 85	118 657 (20.3%)
	+85	38 110 (6.5%)
Race	White	309 646 (52.9%)
	Black	31 872 (5.4%)
	Asian	7108 (1.2%)
	Individuals with multiracial background	235 108 (40.1%)
	Indigenous	1921 (0.3%)
Geographic zone	Urban	557 571 (95.2%)
	Rural	26 215 (4.5%)
	Peri-urban	1869 (0.3%)
Lived in a place with a Flu outbreak†	Yes	121 079 (20.7%)
	No	464 576 (79.3%)
Nosocomial infection	Yes	11 291 (1.9%)
	No	574 364 (98.1%)
**Clinical symptoms**		
Fever	Yes	412 083 (70.4%)
	No	173 572 (29.6%)
Cough	Yes	463 476 (79.1%)
	No	122 179 (20.9%)
Sore throat	Yes	178 618 (30.5%)
	No	407 037 (69.5%)
Dyspnoea	Yes	471 959 (80.6%)
	No	113 696 (19.4%)
Respiratory discomfort	Yes	427 694 (73.0%)
	No	157 961 (27.0%)
Oxygen saturation	<95%	427 152 (72.9%)
	≥95%	158 503 (27.1%)
Diarrhoea	Yes	155 385 (26.5%)
	No	430 270 (73.5%)
Vomit	Yes	118 270 (20.2%)
	No	467 385 (79.8%)
Abdominal pain	Yes	104 898 (17.9%)
	No	480 757 (82.1%)
Fatigue	Yes	212 913 (36.4%)
	No	372 742 (63.6%)
Loss of smell	Yes	144 563 (24.7%)
	No	441 092 (75.3%)
Loss of taste	Yes	146 228 (25.0%)
	No	439 427 (75.0%)
Other symptoms	Yes	264 850 (45.2%)
	No	320 805 (54.8%)
Comorbidities (any)	Yes	389 743 (66.5%)
	No	195 912 (33.5%)
Cardiopathy	Yes	263 365 (45.0%)
	No	322 290 (55.0%)
Hematologic disorder	Yes	6056 (1.0%)
	No	579 599 (99.0%)
Down syndrome	Yes	1732 (0.3%)
	No	583 923 (99.7%)
Hepatic disorder	Yes	8532 (1.5%)
	No	577 123 (98.5%)
Asthma	Yes	46 334 (7.9%)
	No	539 321 (92.1%)
Diabetes mellitus	Yes	218 758 (37.4%)
	No	366 897 (62.6%)
Neurological disorder	Yes	60 654 (10.4%)
	No	525 001 (89.6%)
Chronic respiratory disease	Yes	65 293 (11.1%)
	No	520 362 (88.9%)
Immunosuppressive disorder	Yes	45 652 (7.8%)
	No	540 003 (92.8%)
Renal disease	Yes	67 667 (11.6%)
	No	517 988 (88.4%)
Obesity	Yes	111 219 (19.0%)
	No	474 436 (81.0%)
Other comorbidities†	Yes	236 839 (40.4%)
	No	348 816 (59.6%)
Antiviral drug to treat the infection	Yes	78 153 (13.3%)
	No	507 502 (86.7%)
Need for intensive care unit	Yes	212 067 (36.2%)
	No	373 588 (63.8%)
Need for mechanical ventilatory support	No	141 104 (24.1%)
	No invasive	318 032 (54.3%)
	Invasive	126 519 (21.6%)
Discharge criterion	Laboratorial criterion	548 600 (93.7%)
	Clinical criterion	37 055 (6.3%)
Outcome	Clinical recovery	366 517 (62.6%)
	Death	219 138 (37.4%)
Length of hospital stay		10.85 ± 11.91 d
Length of stay in the intensive care unit		3.51 ± 7.25 d

COVID-19 – Coronavirus Disease 2019, N – number of individuals, SARS-CoV-2 – severe acute respiratory syndrome 2, % – percentage

*We retrieved the individuals’ data from the Brazilian Ministry of Health (https://opendatasus.saude.gov.br/) platform and corresponded to one year of the pandemic (from February 22, 2020, to April 04, 2021). We presented the data as the number of individuals (percentage) or mean ± standard deviation.

†Individuals who lived in an endemic region for Flu; †the “other comorbidities” category did not contain the other comorbidities described in the table.

### Demographic data, clinical signs, comorbidities, and outcomes of Brazilian individuals with SARS due to SARS-CoV-2 infection in Brazil

**Figure 3:**
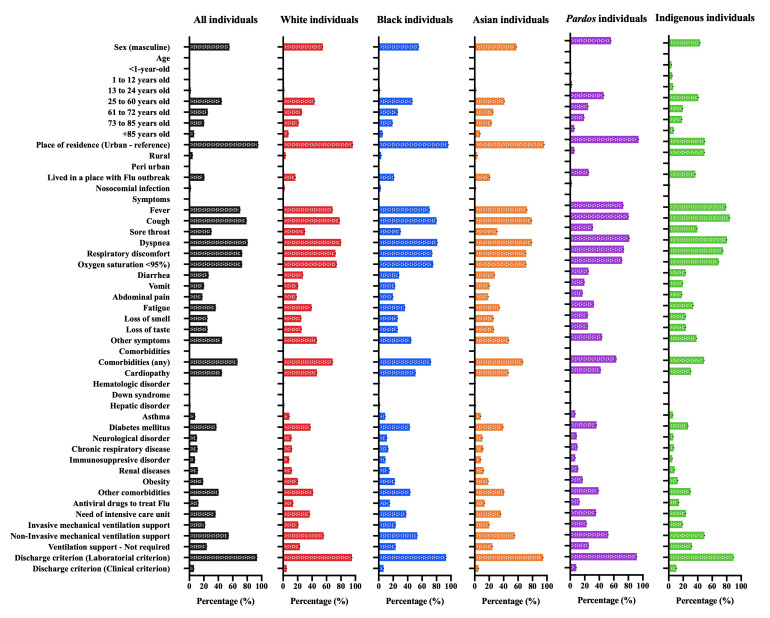
Percentage of the total hospitalized individuals due to Coronavirus Disease (COVID)-19 according to demographic data, clinical signs, comorbidities, and outcomes in all the five Brazilian official races. We retrieved the individuals’ data from the Brazilian Ministry of Health (https://opendatasus.saude.gov.br/) platform and corresponded to one year of the pandemic (from February 22, 2020, to April 04, 2021). We presented the complete information in Supplementary Material.

Most individuals were male (324 210 individuals; 55.4%), aged 25 and above (566 799; 96.9%), lived in urban areas (557 571; 95.2%), and on places with no Flu outbreak (464 576; 79.3%). The nosocomial infection occurred in 11 291 individuals (1.9%) (**Table 1**; **Figure 3**; Table S4 in the **Online Supplementary Document**). We observed a total of 12 clinical symptoms and the most frequent ones were dyspnoea (471 959; 80.6%), cough (463 476; 79.1%), oxygen saturation <95% (427 152; 72.9%), respiratory discomfort (427 694; 73.0%), and fever (412 083; 70.4%) (**Table 1**; **Figure 3**; Table S5 in the **Online Supplementary Document**). Among the 12 comorbidities evaluated in our data, the most frequent were the presence of cardiopathy (263 365; 45.0%), diabetes mellitus (218 758; 37.4%), obesity (111 219; 19.0%), chronic respiratory disease (65 293; 11.1%), and chronic neurological disorder (60 654; 10.4%) (**Table 1**; **Figure 3**; Table S6 in the **Online Supplementary Document**). Also, at least one comorbidity was screened in 389 743 individuals (66.5%) (**Table 1**; **Figure 3**).

The antiviral drug was used only to treat 78 153 (13.3%) individuals. The need for ICUs treatment occurred in 212 067 (36.2%) individuals; also, most individuals needed invasive (126 519; 21.6%) or no invasive (318 032; 54.3%) mechanical ventilation support (**Table 1**; **Figure 3**; Table S7 in the **Online Supplementary Document**). The discharge criterium was first associated with laboratorial findings (548 600; 93.7%). The death occurred in 219 138 (37.4%) individuals (**Table 1**; **Figure 3**; Table S7 in the **Online Supplementary Document**), with a length of hospital stay of 10.85 ± 11.91 days and a length of stay in the ICU of 3.51 ± 7.25 days (**Table 1**).

### Demographic data, clinical signs, comorbidities, and outcomes of Brazilian individuals with SARS due to SARS-CoV-2 infection regarding the five official races in Brazil

**Figure 4:**
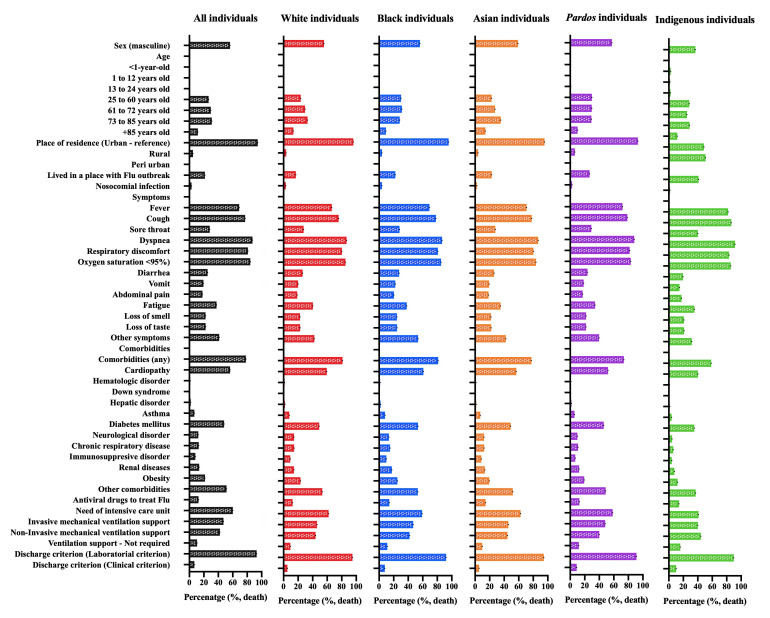
Percentage of the total hospitalized individuals who died due to Coronavirus Disease (COVID)-19 according to demographic data, clinical signs, comorbidities, and outcomes in all the five Brazilian official races. We retrieved the individuals’ data from the Brazilian Ministry of Health (https://opendatasus.saude.gov.br/) platform and corresponded to one year of the pandemic (from February 22, 2020, to April 04, 2021). We presented the complete information in Supplementary Material.

Most White individuals were male (169 535; 54.8%), aged 25 and above (302 396; 97.6%), lived in urban areas (298 145; 96.3%), and on places with no Flu outbreak (257 260; 83.1%). The nosocomial infection occurred in 5902 (1.9%) individuals (Table S8 in the **Online Supplementary Document**; **Figure 3**). We observed a total of 12 clinical symptoms and the most frequent ones were dyspnoea (247 605; 80.0%), cough (242 091; 78.2%), oxygen saturation <95% (229 185; 74.0%), respiratory discomfort (224 218; 72.4%), and fever (211 587; 68.3%) (Table S9 in the **Online Supplementary Document**; **Figure 3**). Among 12 comorbidities, the most frequent were the presence of cardiopathy (144 733; 46.7%), diabetes mellitus (116 606; 37.7%), obesity (62 592; 20.2%), chronic respiratory disease (37 107; 12.0%), renal disease (26 597; 11.8%), and neurological disease (35 324; 11.4%) (Table S10 in the **Online Supplementary Document**; **Figure 3**). The antiviral drug was used only to treat 42 468 (13.7%) individuals. The need for ICUs treatment occurred in 113 594 (36.7%) cases; also, most individuals needed non-invasive (173 507; 56.0%) or invasive (64 499; 20.8%) mechanical ventilation support (Table S11 in the **Online Supplementary Document**; **Figure 3**). The discharge criterium was first associated with the presence of laboratorial findings (294 780; 95.2%) (Table S11 in the **Online Supplementary Document**; **Figure 3**). Death occurred in 110 495 (35.7%) individuals (Table S11 in the **Online Supplementary Document**; **Figure 3**). We also presented the frequency of events in individuals who died only in **Figure 4** and Tables S8 to S11 in the **Online Supplementary Document**.

Among the Black individuals, the most frequent sex was the male ones (17 634; 55.3%), also the individuals aged 25 and above (31 022; 97.3%), lived in urban areas (30 639; 96.1%), and on places with no Flu outbreak (25 126; 78.8%). The nosocomial infection occurred in 863 (2.7%) individuals (Table S12 in the **Online Supplementary Document**; **Figure 3**). We observed a total of 12 clinical symptoms and the most frequent ones were dyspnoea (25 845; 81.1%), cough (25 527; 80.1%), oxygen saturation <95% (23 986; 75.3%), respiratory discomfort (23 596; 74.0%), and fatigue (11 584; 36.3%) (Table S13 in the **Online Supplementary Document**; **Figure 3**). Among 12 comorbidities, the most frequent were the presence of cardiopathy (12 214; 50.9%), diabetes mellitus (13 711; 43.0%), obesity (7089; 22.2%), renal disease (4687; 14.7%), chronic respiratory disease (4128; 11.4%), and neurological disease (3639; 11.4%) (Table S14 in the **Online Supplementary Document**; **Figure 3**). The antiviral drug was used only to treat 4874 (15.3%) individuals. The need for ICUs treatment occurred in 12 106 (38.0%) cases; also, most individuals needed non-invasive (17 026; 53.4%) and invasive (7462; 23.4%) mechanical ventilation support (Table S15 in the **Online Supplementary Document**; **Figure 3**). The discharge criterium was first associated with the presence of laboratorial findings (29 748; 93.3%). Death occurred in 13 289 (41.7%) individuals (Table S15 in the **Online Supplementary Document**; **Figure 3**). We also presented the frequency of events in individuals who died only in **Figure 4** and Tables S12 to S15 in the **Online Supplementary Document**.

Most Asian individuals were male (4102; 57.7%), aged 25 and above (6918; 97.7%), lived in urban areas (6834; 91.1%), and on places with no Flu outbreak (5614; 79.0%). The nosocomial infection occurred in 115 (1.6%) individuals (Table S16 in the **Online Supplementary Document**; **Figure 3**). We observed a total of 12 clinical symptoms and the most frequent ones were dyspnoea (5610; 78.9%), cough (5607; 78.9%), oxygen saturation <95% (5062; 71.2%), respiratory discomfort (5054; 71.1%), and fatigue (2428; 34.2%) (Table S17 in the **Online Supplementary Document**; **Figure 3**). Among 12 comorbidities, the most frequent ones were the presence of cardiopathy (3304; 46.5%), diabetes mellitus (2792; 39.3%), obesity (1330; 18.7%), renal disease (879; 12.4%), chronic respiratory disease (835; 11.7%), and neurological disease (765; 10.8%) (Table S18 in the **Online Supplementary Document**; **Figure 3**). The antiviral drug was used only to treat 982 (13.8%) individuals. The need for ICUs treatment occurred in 2572 (36.2%) cases; also, most individuals needed non-invasive (3938; 55.4%) mechanical ventilation support, followed by 1744 (24.5%) individuals that didn’t require any ventilatory support (Table S19 in the **Online Supplementary Document**; **Figure 3**). The discharge criterium was first associated with laboratorial findings (6723; 94.6%). Death occurred in 2532 (35.6%) individuals. We also presented the frequency of events in individuals who died only in **Figure 4** and Tables S16 to S19 in the **Online Supplementary Document**.

Among the multiracial background individuals, the most frequent sex was male (131 850; 56.1%), also the individuals aged 25 and above (224 810; 95.6%), lived in urban areas (220 996; 94.0%), and on places with no Flu outbreak (175 360; 74.6%). The nosocomial infection occurred in 4385 (1.9%) individuals (Table S20 in the **Online Supplementary Document**; **Figure 3**). We observed a total of 12 clinical symptoms and the most frequent ones were dyspnoea (191 356; 81.4%), cough (188 633; 80.2%), respiratory discomfort (173 386; 73.7%), oxygen saturation <95% (167 594; 71.3%), and fatigue (76 274; 32.4%) (Table S21 in the **Online Supplementary Document**; **Figure 3**). Among 12 comorbidities, the most frequent were the presence of cardiopathy (98 524; 41.9%), diabetes mellitus (85 136; 36.2%), obesity (39 971; 17.0%), renal disease (25 348; 10.8%), chronic respiratory disease (23 088; 9.8%), and neurological disease (20 806; 8.8%) (Table S22 in the **Online Supplementary Document**; **Figure 3**). The antiviral drug was used only to treat 29 562 (12.6%) individuals. The need for ICUs treatment occurred in 83 339 (35.4%) cases; also, most individuals needed non-invasive (122 615; 52.2%) mechanical ventilation support, followed by 59 726 (25.4%) that didn’t require any type of ventilatory support (Table S23 in the **Online Supplementary Document**; **Figure 3**). The discharge criterium was first associated with the presence of laboratorial findings (215 632; 91.7%). Death occurred in 92 049 (39.1%) individuals (Table S23 in the **Online Supplementary Document**; **Figure 3**). We also presented the frequency of events in individuals who died only in **Figure 4** and Tables S20 to S23 in the **Online Supplementary Document**.

Finally, most Indigenous peoples were female (1089; 56.7%), aged 25 and above (1642; 85.5%), lived in urban areas (957; 49.2%); and on places with no Flu outbreak (1216; 63.3%) (Table S24 in the **Online Supplementary Document**; **Figure 3**). We observed a total of 12 clinical symptoms and the most frequent ones were cough (1618; 84.2%), dyspnoea (1543; 80.3%), respiratory discomfort (1440; 75.0%), oxygen saturation <95% (1325; 69.0%), and fatigue (649; 33.8%) (Table S25 in the **Online Supplementary Document**; **Figure 3**). Among 12 comorbidities, the most frequent were the presence of cardiopathy (590; 30.7%), diabetes mellitus (513; 26.7%), obesity (237; 12.3%), renal disease (156; 8.1%), chronic respiratory disease (135; 7.0%), and neurological disease (120; 6.2%) (Table S26 in the **Online Supplementary Document**; **Figure 3**). The antiviral drug was used only to treat 268 (14.0%) individuals. The need for ICUs treatment occurred in 456 (23.7%) cases; also, most individuals required non-invasive (946; 49.2%) mechanical ventilation support, followed by 610 (31.8%) individuals that didn’t require any ventilatory support (Table S27 in the **Online Supplementary Document**; **Figure 3**). The discharge criterium was first associated with laboratorial findings (1717; 89.4%). The death occurred in 773 (40.2%) individuals (Table S27 in the **Online Supplementary Document**; **Figure 3**). We also presented the frequency of events in individuals who died only in **Figure 4** and Tables S24 to S27 in the **Online Supplementary Document**.

### Race as a risk factor of death due to COVID-19 in Brazilian individuals with SARS according to the mechanical ventilatory support

A higher proportion of overall case fatality rate occurred in Black individuals (OR = 1.29; 95% CI = 1.26-1.32), individuals with multiracial backgrounds (OR = 1.16; 95% CI = 1.15-1.17), and Indigenous peoples (OR = 1.21; 95% CI = 1.11-1.33) compared to White individuals. Also, a higher case fatality rate occurred in Black individuals (OR = 1.50; 95% CI = 1.41-1.59), individuals with multiracial backgrounds (OR = 1.28; 95% CI = 1.30-1.38), and Indigenous peoples (OR = 1.49; 95% CI = 1.21-1.81) compared to White individuals who did not need any ventilatory support. In individuals who needed non-invasive ventilatory support, higher mortality was observed in Black individuals (OR = 1.25; 95% CI = 1.20-1.29), individuals with multiracial backgrounds (OR = 1.10; 95% CI = 1.08-1.12), and Indigenous peoples (OR = 1.45; 95% CI = 1.27-1.66). Regarding individuals who needed invasive ventilatory support, a higher case fatality rate was observed in Black individuals (OR = 1.29; 95% CI = 1.21-1.37), individuals with multiracial backgrounds (OR = 1.31; 95% CI = 1.27-1.35), and Indigenous peoples (OR = 1.39; 95% CI = 1.05-1.85) (**Table 2**). No statistical difference occurred for the Asian race compared to White individuals.

**Table 2:** Odds ratio (OR) of death in hospitalized Coronavirus Disease (COVID)-19 Brazilian individuals regarding the mechanical ventilatory support*

Ventilatory support	Race	Death	Recovery	Total	*P*	OR (95%CI)
Invasive mechanical ventilation	White	51 520 (49.8%)	12 979 (56.3%)	64 499 (51.0%)		Reference
	Black	6239 (6.0%)	1223 (5.3%)	7462 (5.9%)	<0.001	1.285 (1.205-1.370)
	Asian	1156 (1.1%)	270 (1.2%)	1426 (1.1%)	0.284	1.079 (0.945-1.233)
	Individuals with multiracial background	44 254 (42.8%)	8513 (36.9%)	52 767 (41.7%)	<0.001	1.310 (1.271-1.350)
	Indigenous	309 (0.2%)	56 (0.2%)	365 (0.3%)	0.027	1.390 (1.045-1.849)
Non-invasive mechanical ventilation	White	48 656 (52.6%)	124 851 (55.4%)	173 507 (54.6%)		Reference
	Black	5563 (6.0%)	11 463 (5.1%)	17 026 (5.4%)	<0.001	1.245 (1.204-1.288)
	Asian	1127 (1.2%)	2811 (1.2%)	3938 (1.2%)	0.437	1.029 (0.959-1.103)
	Individuals with multiracial background	36 796 (39.8%)	85 819 (38.0%)	122 615 (38.6%)	<0.001	1.100 (1.083-1.118)
	Indigenous	342 (0.4%)	604 (0.3%)	946 (0.3%)	<0.001	1.453 (1.272-1.660)
None	White	10 319 (44.5%)	61 321 (52.0%)	71 640 (50.8%)		Reference
	Black	1487 (6.4%)	5897 (5.0%)	7384 (5.2%)	<0.001	1.498 (1.410-1.592)
	Asian	249 (1.1%)	1495 (1.3%)	1744 (1.2%)	0.909	0.990 (0.864-1.134)
	Individuals with multiracial background	10 999 (47.5%)	48 727 (41.3%)	59 726 (42.3%)	<0.001	1.279 (1.303-1.381)
	Indigenous	122 (0.5%)	488 (0.4%)	610 (0.4%)	<0.001	1.486 (1.217-1.814)
All individuals independently of the ventilatory support	White	110 495 (50.4%)	199 151 (54.3%)	309 646 (52.9%)		Reference
	Black	13 289 (6.1%)	18 583 (5.1%)	31 872 (5.4%)	<0.001	1.289 (1.254-1.319)
	Asian	2532 (1.2%)	4576 (1.2%)	7108 (1.2%)	0.923	0.997 (0.950-1.047)
	Individuals with multiracial background	92 049 (42.0%)	143 059 (39.0%)	235 108 (40.1%)	<0.001	1.160 (1.147-1.173)
	Indigenous	773 (0.4%)	1148 (0.3%)	1921 (0.3%)	<0.001	1.214 (1.108-1.330)

95% CI – 95% confidence interval

*We retrieved the individuals’ data from the Brazilian Ministry of Health (https://opendatasus.saude.gov.br/) platform and corresponded to one year of the pandemic (from February 22, 2020, to April 04, 2021). We presented the data as the number of individuals (N) and the percentage (%).

### Characteristics associated with the case fatality rate due to COVID-19 in Brazilian individuals with SARS

**Figure 5:**
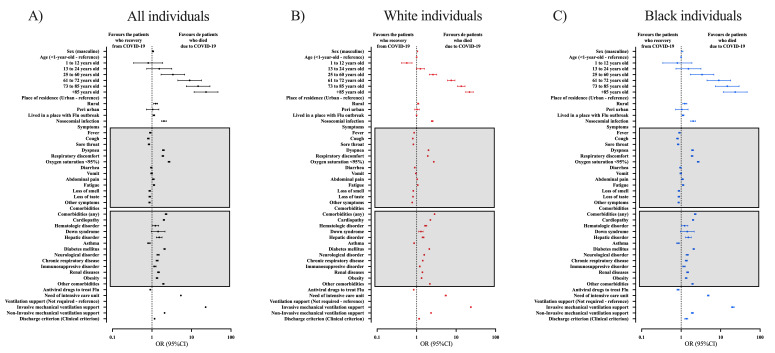
Bivariate analyses to determine the individuals’ characteristics that contributed to the risk of death. We presented the races as colours: A) all individuals hospitalized due to Coronavirus Disease (COVID)-19 in Brazil; B) White individuals hospitalized due to COVID-19 in Brazil; C) Black individuals hospitalized due to COVID-19 in Brazil. We presented the data as odds ratio (OR) and 95% confidence interval (95% CI). We retrieved the individuals’ data from the Brazilian Ministry of Health (https://opendatasus.saude.gov.br/) platform and corresponded to one year of the pandemic (from February 22, 2020, to April 04, 2021). We presented the complete information in Supplementary Material.

The association between the case fatality rate due to SARS-CoV-2 infection and demographic characteristics of all the individuals demonstrated a higher OR for death in male individuals (OR = 1.07; 95% CI = 1.06-1.08) compared to females and in older individuals (73 to 85 years old (OR = 10.30, 95% CI = 9.17-11.56); +85 years old (OR = 16.16; 95% CI = 14.38-18.17)) compared to <1-year-old individuals. Also, other essential factors associated with the enhanced OR of death were people who lived in a rural zone (OR = 1.20; 95% CI = 1.17-1.24) and nosocomial infection (OR = 2.17; 95% CI = 2.09-2.25) (Table S4 in the **Online Supplementary Document**). All clinical symptoms demonstrated a significant association with case fatality rate except the presence of fatigue. Also, among the clinical symptoms, we obtained a higher OR for oxygen saturation <95% (OR = 2.75; 95% CI = 2.72-2.79), dyspnoea (OR = 2.09; 95% CI = 2.06-2.12), and respiratory discomfort (OR = 1.97; 95% CI = 1.94-1.99), most of the other symptoms were more frequent in those who survived (Table S5 in the **Online Supplementary Document**). In addition, all comorbidities contributed as a risk factor of death among the individuals with COVID-19, mainly the presence of cardiopathy (OR = 2.088; 95% CI = 2.06-2.11), diabetes mellitus (OR = 2.05; 95% CI = 2.02-2.07), and hematologic disorder (OR = 1.52; 95% CI = 1.45-1.60) (Table S6 in the **Online Supplementary Document**). The hospitalized individuals who died due to COVID-19 needed more ICU treatment (OR = 5.39; 95% CI = 5.33-5.45); and invasive (OR = 22.85; 95% CI = 22.40-23.31) or non-invasive mechanical ventilation support (OR = 2.08; 95% CI = 2.05-2.12) (Table S7 in the **Online Supplementary Document**). We also presented the information in **Figure 5**, Panel A.

Regarding the demographic characteristics in White individuals associated with case fatality rate, we observed male (OR = 1.05; 95% CI = 1.03-1.07), age (73 to 85 years old (OR = 13.51; 95% CI = 10.85-16.82) and +85 years old (OR = 21.82; 95% CI = 17.51-27.19)), people who lived in rural areas (OR = 1.09; 95% CI = 1.04-1.13), and nosocomial infection (OR = 2.47; 95% CI = 2.35-2.61) to risk factors for death (Table S8 in the **Online Supplementary Document**). White individuals presenting symptoms such as oxygen saturation <95% (OR = 2.71; 95% CI = 2.66-2.76), dyspnoea (OR = 1.99; 95% CI = 1.95-2.03), and respiratory discomfort (OR = 1.91; 95% CI = 1.88-1.94) (Table S9 in the **Online Supplementary Document**); and comorbidities such as cardiopathy (OR = 2.22; 95% CI = 2.19-2.25), diabetes mellitus (OR = 2.09; 95% CI = 2.06-2.12), and hematologic disorder (OR = 1.69; 95% CI = 1.58-1.81) also presented an enhanced risk of death (Table S10 in the **Online Supplementary Document**). White individuals who died presented a higher frequency of need for ICU (OR = 5.52; 95% CI = 5.23-5.61), invasive (OR = 23.59; 95% CI = 22.93-24.27) and non-invasive (OR = 2.31; 95% CI = 2.262-2.37) mechanical ventilatory support (Table S11 in the **Online Supplementary Document**). We also presented the information in **Figure 5**, Panel B.

In Black individuals, we observed some demographic characteristics to be associated with enhanced risk of death, such as male (OR = 1.06; 95% CI = 1.01-1.10), older age (73 to 85 years old (OR = 14.71; 95% CI = 7.37-29.34) and +85 years old (OR = 23.38; 95% CI = 11.66-49.90)), those who lived in urban areas (OR = 1.24; 95% CI = 1.10-1.39) and nosocomial infection (OR = 2.01; 95% CI = 1.75-2.30) (Table S12 in the **Online Supplementary Document**). Those who presented symptoms such as oxygen saturation <95% (OR = 2.70; 95% CI = 2.55-2.86), dyspnoea (OR = 1.94; 95% CI = 1.82-2.06), and respiratory discomfort (OR = 1.89; 95% CI = 1.79-1.99) (Table S13 in the **Online Supplementary Document**), and some comorbidities such as diabetes mellitus (OR = 2.08; 95% CI = 1.98-2.17), cardiopathy (OR = 2.00; 95% CI = 1.91-2.39), and hepatic disorder (OR = 1.52; 95% CI = 1.28-1.80) were at higher risk of death (Table S14 in the **Online Supplementary Document**). ICU treatment (OR = 4.85; 95% CI = 4.62-5.10), need for invasive (OR = 20.23; 95% CI = 18.61-21.99) and non-invasive (OR = 1.92; 95% CI = 1.803-2.05) mechanical ventilatory support were also risk factors of death (Table S15 in the **Online Supplementary Document**). We also presented the information in **Figure 5**, Panel C.

**Figure 6:**
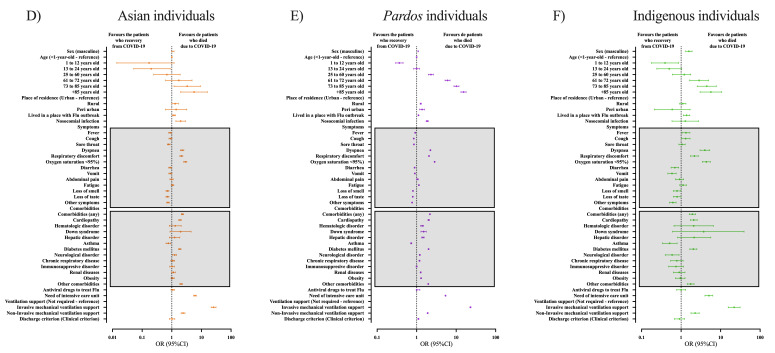
Bivariate analyses to determine the individuals’ characteristics that contributed to the risk of death. We presented the races as colours: A) Asian individuals hospitalized due to COVID-19 in Brazil; B) multiracial backgrounds (*Pardos*) individuals hospitalized due to COVID-19 in Brazil; and C) Indigenous peoples hospitalized due to COVID-19 in Brazil. We presented the data as odds ratio (OR) and 95% confidence interval (95% CI). We retrieved the individuals’ data from the Brazilian Ministry of Health (https://opendatasus.saude.gov.br/) platform and corresponded to one year of the pandemic (from February 22, 2020, to April 04, 2021). We presented the complete information in Supplementary Material.

In Asian individuals, only age (73 to 85 years old (OR = 3.31; 95% CI = 1.18-9.23) and +85 years old (OR = 5.64; 95% CI = 2.00-15.93)), and nosocomial infection (OR = 1.99; 95% CI = 1.37-2.88) were associated with enhanced mortality as demographic characteristics (Table S16 in the **Online Supplementary Document**). Symptoms like oxygen saturation <95% (OR = 2.85; 95% CI = 2.53-3.22), dyspnoea (OR = 2.23; 95% CI = 1.95-2.55), and respiratory discomfort (OR = 2.10; 95% CI = 1.87-2.36) (Table S17 in the **Online Supplementary Document**); and comorbidities such as cardiopathy (OR = 1.89; 95% CI = 1.72-2.09), diabetes mellitus (OR = 1.80; 95% CI = 1.68-2.05), and neurological disorder (OR = 1.24; 95% CI = 1.07-1.45) were more present in those who died (Table S18 in the **Online Supplementary Document**). Asian individuals who needed ICU treatment (OR = 6.06; 95% CI = 5.45-6.75), and need invasive (OR = 25.71; 95% CI = 21.29-31.04) or non-invasive (OR = 2.40; 95% CI = 2.07-2.79) mechanical ventilatory support were more prone to die (Table S19 in the **Online Supplementary Document**). We also presented the information in **Figure 6**, Panel A.

Individuals with multiracial background showed several demographic characteristics as a risk factor of death, such as male sex (OR = 1.09; 95% CI = 1.07-1.11), older age (73 to 85 years old (OR = 10.170; 95% CI = 8.77-11.78) and +85 years old (OR = 15.37; 95% CI = 13.22-17.87)), place of residence in a rural (OR = 1.26; 95% CI = 1.22-1.31) or peri-urban (OR = 1.37; 95% CI = 1.19-1.57), and nosocomial infection (OR = 1.85; 95% CI = 1.74-1.96) (Table S20 in the **Online Supplementary Document**). Those who presented symptoms like oxygen saturation <95% (OR = 2.84; 95% CI = 2.78-2.90), dyspnoea (OR = 2.22; 95% CI = 2.17-2.28), and respiratory discomfort (OR = 2.05; 95% CI = 2.1-2.09) (Table S21 in the **Online Supplementary Document**); and comorbidities such as diabetes mellitus (OR = 2.00 95% CI = 1.96-2.03), cardiopathy (OR = 1.98; 95% CI = 1.94-2.10), and Down syndrome (OR = 1.50; 95% CI = 1.28-1.74) presented an enhanced risk of death (Table S22 in the **Online Supplementary Document**). Those who needed ICU treatment (OR = 5.38; 95% CI = 5.28-5.48), invasive (OR = 23.03; 95% CI = 22.32-23.76) or non-invasive (OR = 1.89; 95% CI = 1.85-1.94) mechanical ventilatory support also presented a higher risk of death (Table S23 in the **Online Supplementary Document**). We also presented the information in **Figure 6**, Panel B.

Indigenous individuals who were male (OR = 1.57; 95% CI = 1.30-1.89) and older age (73 to 85 years old (OR = 4.47; 95% CI = 2.55-7.81) and +85 years old (OR = 5.67; 95% CI = 3.02-10.68)) also presented a higher risk of death (Table S24 in the **Online Supplementary Document**). Symptoms like oxygen saturation <95% (OR = 4.36; 95% CI = 3.45-5.50), dyspnoea (OR = 3.99; 95% CI = 3.01-5.31), and respiratory discomfort (OR = 2.17; 95% CI = 1.73-2.73) (Table S25 in the **Online Supplementary Document**); and comorbidities such as cardiopathy (OR = 2.08; 95% CI = 1.72-2.55) (Table S26 in the **Online Supplementary Document**) were more frequent in individuals who died. Individuals who needed ICU treatment (OR = 5.04; 95% CI = 4.01-6.33), invasive (OR = 22.07; 95% CI = 15.60-31.23), or non-invasive (OR = 2.26 95% CI = 1.78-2.87) mechanical ventilatory support also were at increased risk of death (Table S27 in the **Online Supplementary Document**). We also presented the information in **Figure 6**, Panel C.

### Time of hospitalization and the need for ICU in hospitalized Brazilian individuals with SARS according to the race

**Figure 7:**
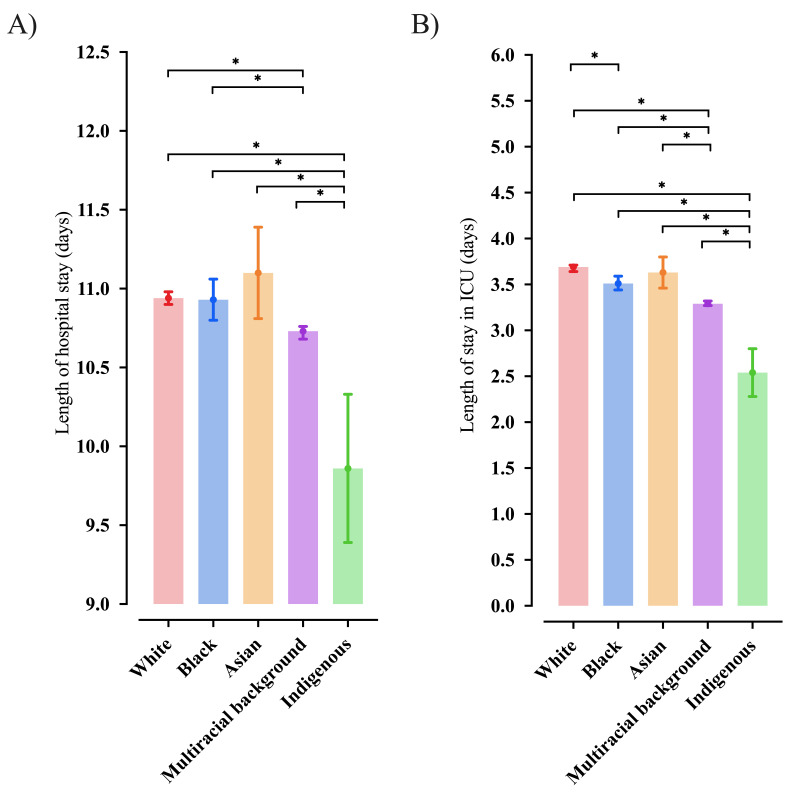
Length of hospital stay (days) and length of stay in the intensive care unit ICU (days) according to the races of hospitalized individuals due to severe acute respiratory syndrome (SARS) due to Coronavirus Disease (COVID)-19 in Brazil. *, *P* was significative (<0.05). We presented the data as mean and 95% confidence interval (95%CI). We retrieved the individuals’ data from the Brazilian Ministry of Health (https://opendatasus.saude.gov.br/) platform and corresponded to one year of the pandemic (from February 22, 2020, to April 04, 2021). We presented the complete information in Supplementary Material.

According to the race, we presented the association between the length of hospital stay (**Figure 7**, Panel A) and the length of stay in the ICU (**Figure 7**, Panel B). In our data, the individuals identified as Indigenous presented a lower number of days for the length of hospital and length of stay in the ICU than the other races. Also, individuals identified as multiracial background had a lower number of days in the hospital than those identified as White and Black; in addition, the same group of individuals had a low number of days in the ICU than those White, Black, and Asian. Finally, individuals identified as Black stayed a low number of days in the ICU than White ones.

### Multivariate analysis to determine the risk of death due to COVID-19 in Brazilian individuals with SARS

Our multivariate model using the Logistic Regression (Backward model) was able to predict (78.6%) the death due to SARS-CoV-2 infection (χ^2^ = 380.775; df = 8; *P* < 0.001; R^2^ Nagelkerke = 0.452). We did not include the comorbidities (“any” or “other” categories) and symptoms (other) from the individuals’ characteristics. Also, the location of residence in a region with a Flu outbreak, fever, fatigue, and ICU length from the analysis was not significant, and we removed them using the backward stepwise model. The race was positive and significant in predicting death. Black (OR = 1.43; 95% CI = 1.39-1.48) people, individuals with multiracial background (OR = 1.36; 95% CI = 1.34-1.38), and Indigenous (OR = 1.91; 95% CI = 1.70-2.15) peoples were more prone to die compared to the White race. The Asian individuals did not have a higher chance of dying due to SARS-CoV-2 infection than White individuals (OR = 0.94; 95% CI = 0.99-1.06). In addition, other individuals’ characteristics had a higher frequency in individuals who died due to COVID-19, such as male (OR = 1.17; 95% CI = 1.16-1.19), age (13 to 24 years old (OR = 1.36; 95% CI = 1.17-1.58), 25 to 60 years old (OR = 2.39; 95% CI = 2.08-2.73), 61 to 72 years old (OR = 5.94; 95% CI = 5.19-6.81), 73 to 85 years old (OR = 11.43; 95% CI = 9.98-13.11), and +85 years old (OR = 23.02; 95% CI = 20.05-26.42) compared to 1-year-old group), living in rural areas (OR = 1.22; 95% CI = 1.18-1.26) or in peri-urban places (OR = 1.25; 95% CI = 1.11-1.40), and the presence of nosocomial infection (OR = 1.91; 95% CI = 1.82-2.01). Among the clinical symptoms, the most frequent ones among the individuals who died due to COVID-19 were dyspnoea (OR = 1.25; 95% CI = 1.23-1.28), respiratory discomfort (OR = 1.30; 95% CI = 1.28-1.32), and oxygen saturation <95% (OR = 1.40; 95% CI = 1.38-1.43). Also, among the comorbidities, the most frequent ones among the individuals who died due to COVID-19 were cardiopathy (OR = 1.13; 95% CI = 1.11-1.14), hematologic disorder (OR = 1.34; 95% CI = 1.24-1.43), Down syndrome (OR = 1.61; 95% CI = 1.43-1.81), hepatic disorder (OR = 1.41; 95% CI = 1.34-1.50), diabetes mellitus (OR = 1.40; 95% CI = 1.37-1.42), neurological disorder (OR = 1.21; 95% CI = 1.17-1.25), immunosuppressive disorder (OR = 1.44; 95% CI = 1.39-1.49), renal disease (OR = 1.15; 95% CI = 1.11-1.18), and obesity (OR = 1.18; 95% CI = 1.15-1.21). Hospitalized individuals who needed ICUs treatment (OR = 2.25; 95% CI = 2.22-2.29) and who needed mechanical ventilation (invasive (OR = 10.92; 95% CI = 10.66-11.18) and non-invasive (OR = 1.33; 95% CI = 1.30-1.35)) had a higher chance of death; also, among this group of individuals, the clinical discharge criterium was more frequent (OR = 1.09; 95% CI = 1.06-1.12) (**Table 3**).

**Table 3:** Multivariate analysis by logistic regression (Backward method) to determine the patients’ characteristics that contributed to the risk of death*

Markers	B	SE	Wald	df	*P*-value	OR	95% CI	
**Lower**	**Upper**	
Sex (male)	0.158	0.007	526.943	1	<0.001	1.172	1.156	1.187	
**Age**									
<1-y-old			44 628.873	6	<0.001				
1 to 12 y old	-0.570	0.097	34.820	1	<0.001	0.565	0.468	0.683	
13 to 24 y old	0.306	0.078	15.553	1	<0.001	1.359	1.167	1.582	
25 to 60 y old	0.869	0.069	156.679	1	<0.001	2.385	2.081	2.732	
61 to 72 y old	1.782	0.070	656.647	1	<0.001	5.944	5.186	6.812	
73 to 85 y old	2.437	0.070	1 224.340	1	<0.001	11.434	9.976	13.107	
+85 y old	3.136	0.070	1 985.781	1	<0.001	23.017	20.051	26.421	
**Race**									
White			2 120.794	4	<0.001				
Black	0.360	0.015	574.801	1	<0.001	1.433	1.391	1.475	
Asian	-0.001	0.031	0.002	1	0.964	0.999	0.939	1.062	
Multiracial background	0.305	0.007	1 797.757	1	<0.001	1.356	1.337	1.375	
Indigenous	0.648	0.059	119.225	1	<0.001	1.912	1.702	2.148	
**Place of residence**									
Urban			166.812	2	<0.001				
Rural	0.200	0.016	154.125	1	<0.001	1.222	1.184	1.261	
Peri-urban	0.222	0.059	13.955	1	<0.001	1.249	1.111	1.403	
Nosocomial infection	0.649	0.024	708.496	1	<0.001	1.914	1.824	2.007	
**Clinical symptoms**								
Cough	-0.160	0.009	334.333	1	<0.001	0.852	0.837	0.867	
Sore throat	-0.030	0.010	9.729	1	0.002	0.970	0.952	0.989	
Dyspnoea	0.226	0.010	488.417	1	<0.001	1.254	1.229	1.280	
Respiratory discomfort	0.262	0.009	814.625	1	<0.001	1.299	1.276	1.323	
Oxygen saturation (<95%)	0.339	0.010	1 270.580	1	<0.001	1.404	1.378	1.431	
Diarrhoea	-0.097	0.011	77.798	1	<0.001	0.908	0.889	0.928	
Vomit	-0.107	0.013	64.035	1	<0.001	0.899	0.875	0.922	
Abdominal pain	-0.041	0.015	7.340	1	0.007	0.959	0.931	0.989	
Loss of smell	-0.071	0.021	11.882	1	0.001	0.931	0.894	0.970	
Loss of taste	-0.240	0.020	138.046	1	<0.001	0.786	0.755	0.819	
**Comorbidities**								
Cardiopathy	0.118	0.008	204.878	1	<0.001	1.125	1.107	1.144	
Hematologic disorder	0.289	0.034	70.148	1	<0.001	1.335	1.248	1.428	
Down syndrome	0.476	0.060	62.399	1	<0.001	1.609	1.430	1.810	
Hepatic disorder	0.346	0.029	141.430	1	<0.001	1.413	1.335	1.496	
Asthma	-0.750	0.020	1 435.782	1	<0.001	0.472	0.454	0.491	
Diabetes mellitus	0.333	0.009	1 513.319	1	<0.001	1.395	1.372	1.418	
Neurological disorder	0.190	0.016	149.325	1	<0.001	1.209	1.173	1.246	
Chronic respiratory disease	-0.096	0.016	36.161	1	<0.001	0.908	0.880	0.937	
Immunosuppressive disorder	0.365	0.019	374.284	1	<0.001	1.440	1.388	1.494	
Renal disease	0.138	0.016	76.868	1	<0.001	1.148	1.113	1.184	
Obesity	0.167	0.012	180.164	1	<0.001	1.182	1.153	1.211	
Antiviral drugs to treat the infection	-0.203	0.010	400.722	1	<0.001	0.816	0.800	0.833	
Need for intensive care unit	0.814	0.008	10 883.354	1	<0.001	2.256	2.222	2.291	
Need for ventilatory support					<0.001				
None			51 882.806	2	<0.001				
Invasive	2.390	0.012	37 951.425	1	<0.001	10.915	10.655	11.180	
Non-invasive	0.281	0.009	899.980	1	<0.001	1.325	1.301	1.349	
Discharge criterion (clinical)	0.086	0.014	39.463	1	<0.001	1.090	1.061	1.120	
Length of hospital stay (days)	-0.005	<0.001	333.859	1	<0.001	0.995	0.994	0.995	
Constant	-3.925	0.070	3 109.945	1	<0.001	0.020			

In addition, the individuals aged 1 to 12 years old compared to those in the 1-year-old group had a lower chance of death (OR = 0.57; 95% CI = 0.47-0.68). Also, the individuals who died used fewer antiviral drugs (OR = 0.82; 95% CI = 0.89-0.83) and stayed a low number of days in the hospital compared to those who recovered from the SARS-CoV-2 infection (OR = 0.99; 95% CI = 0.99-0.99). The clinical symptoms (cough (OR = 0.85; 95% CI = 0.84-0.87), sore throat (OR = 0.97; 95% CI = 0.95-0.99), diarrhoea (OR = 0.91; 95% CI = 0.89-0.93), vomit (OR = 0.90; 95% CI = 0.88-0.92), abdominal pain (OR = 0.96; 95% CI = 0.93-0.99), loss of smell (OR = 0.93; 95% CI = 0.89-0.97, and (OR = 0.79; 95% CI = 0.76-0.82)) were less prevalent among the individuals who died compared to those who recovered from the COVID-19 (**Table 3**). We presented the detailed analysis in **Table 3**.

## DISCUSSION

In our study, several demographic data, clinical symptoms as well as comorbidities were associated with enhanced risk of death due to COVID-19, like older age, male sex, respiratory discomfort, oxygen saturation <95%, dyspnoea, abdominal pain, immunosuppressive disorder, neurological disorder, renal disorder, hepatic disorder, chronic respiratory disease, need for mechanical ventilation and especially race, which is similar to the current literature [5,7,45-47]. Also, we observed that in addition to the race being associated with death itself, it is associated with patients’ characteristics associated with death, suggesting the impact of race on the risk of death to be even higher. In the clinical profile of each race, we observed that Black individuals have more symptoms related to enhanced severity, such as dyspnoea and respiratory discomfort, compared to White individuals.

Unfortunately, most individuals were White in our cohort, with 309 646 (52.9%) individuals. Individuals with multiracial backgrounds accounted for only 235 108 (40.1%) cases, which might not represent the Brazilian scenario since most Brazilians are individuals with multiracial backgrounds and not White [44]. The contradictory race prevalence might reflect the low access to health care experienced by those people [48] or even a bias associated with the race being self-declared, which could mask the actual distribution of races in Brazil.

Several studies tried to evaluate the impact of COVID-19 on different races worldwide, including a meta-analysis. One recent meta-analysis compiled 50 studies that observed a higher infection rate of SARS-CoV-2 and higher ICU admission among Black individuals [15]. However, only Asian individuals were at increased mortality risk. In another meta-analysis, the authors observed that Black individuals were at increased risk of mortality, hospitalization, and ICU admission. In contrast, Asians were more prone to die, and Hispanics were more prone to hospitalization compared to White individuals [16]. In addition, after applying the Grading of Recommendations Assessment, Development and Evaluation (GRADE), the same meta-analysis found a neutral effect of the Black race on COVID-19 mortality [16].

Another study also evaluated the impact of race on COVID-19 and observed a higher burden of the pandemic on Black and Hispanic individuals, with enhanced mortality, infection rate, and hospitalization [49]. Other studies tried to evaluate the impact of COVID-19 on the Indigenous peoples, in which enhanced death was observed in American Indians, Alaska Natives, and Indigenous individuals from Australia [50,51]. Finally, only a few studies evaluated individuals with multiracial backgrounds, and higher mortality and admission to ICU has observed [52,53]. Our study is by the literature and the international studies since we also observed enhanced mortality in Black individuals, Indigenous peoples, and individuals with multiracial backgrounds and enhanced ICU admission and the need for IMV. We did not observe enhanced mortality of Asian individuals; perhaps these individuals have equal access to health care centres as the White population – reference in our analysis.

We observed that races present different symptoms of COVID-19 infections. In this matter, several clinical signs occurred mainly in Indigenous peoples as the presence of cough, dyspnoea, respiratory discomfort, and oxygen saturation <95%. In contrast, individuals with multiracial backgrounds had more respiratory discomfort; Black individuals had more fatigue, and White individuals demonstrated a higher frequency of fever than other races [54-56]. The symptoms described before occurred in individuals with severe COVID-19 [57,58]. Furthermore, we also noticed that most of the other symptoms, such as vomit and diarrhoea, appear to be protective against death; perhaps, individuals who experienced these symptoms rushed to a health facility and received proper treatment, thus preventing death.

A study by McCarty et al. [59] tried a similar approach to how different races developed specific symptoms. In Massachusetts, the study included 379 patients hospitalized for COVID-19, which showed that fever was more common in the Latinx race, fatigue in Asians; dyspnoea, sore throat, cough, myalgia, and chills in Latinx; diaphoresis and Arthralgia in White. The difference between this study’s results from ours can be explained by the lower number of patients selected for the study in Massachusetts, restricting its accuracy. Nonetheless, the differences between Brazil’s and America’s populations need to be pointed out, as different races with diverse historic backgrounds may display other symptoms.

In Brazil, several studies also tried to evaluate the impact of COVID-19 among the different races across all the Brazilian territories [5,7,12,26,34,35]. Peres et al. conducted an observational study comprising more than 222 000 Brazilian hospitalized patients with positive COVID-19 tests, out of which 37% died. The authors observed the Black/Brown race (multiracial background, *Pardos*) as a risk factor for in-hospital death [26]. Rodrigues et al. comprised more than 800 000 hospitalized patients with COVID-19 across all the five Brazilian regions, with the death of nearly 330 000 individuals. Higher in-hospital mortality was observed in Black individuals, especially those from the central and south regions [35].

In the same way, Baqui et al. comprised nearly 90 000 hospitalized patients with COVID-19, and the authors observed a higher death rate in Black and multiracial background (*Pardos*) individuals. In this study, Pardo's race was the second most important risk factor, just behind age [5]. Martins-Filho also found enhanced mortality in Black individuals across the five Brazilian regions. In addition, these individuals from the Central West region seem to be at even higher risk of death [7]. Another study performed by Sansone et al. observed enhanced mortality and an increased burden of COVID-19 in Indigenous peoples [12]. Our results follow previous Brazilian studies.

In our study, the Indigenous peoples experience some unique traits; for instance, most of them affected by COVID-19 were females, not males, like the other races. Perhaps their unique way of living, genetic background, and culture [12] might provide an epidemiological pattern quite different from the other races. Furthermore, to the best of our knowledge, no study tried to describe the clinical profile of each Brazilian race as we did.

The impact of COVID-19 seems to be higher in those from minority groups. Several socioeconomic and demographic traits might be responsible, at least in part, for this higher burden. For instance, Black individuals seem to have a higher prevalence of important comorbidities, such as systemic arterial hypertension, diabetes mellitus, and obesity, which have already been described as associated with mortality [6,10]. Unfortunately, individuals from minority groups with lower job security and low-income experienced job loss [60,61], which might have led them to pursue informal jobs with enhanced exposure risk to COVID-19. Also, those who did not lose their job appear to fill jobs with a higher risk of COVID-19 contagion and exposure, with an inability to work from home, which also jeopardizes them [62,63].

Other socioeconomic factors might also be important in the different case fatality rates experienced by the races; for instance, a recent report showed a negative association between the county median income and COVID-19 case fatality in Black and Hispanic individuals [20]. In the same way, the Human Development Index was also negatively associated with COVID-19 case fatality [27]. Unfortunately, although access to health care centres has increased over the years, individuals from minority groups still have poor access to health [14], and in Brazil, it is no different. Even though one of the three doctrinal principles of the *Sistema Único de Saúde* (SUS; Brazilian Public Healthcare) is equal access to health care for all the citizens [64], the minority groups have low access to health care and face racial discrimination, which can impair their condition of living, overall health and even the willingness to seek health care when needed [48,65].

As in the world, this scenario of disparity is also repeated, affecting mainly Blacks, individuals with multiracial backgrounds, and Indigenous peoples to the detriment of White individuals. Structural racism exposes these minority groups to crowded transportation, housing in crowded houses, and little government incentives for prevention campaigns advocated by the World Health Organization. Understanding and pointing out these weaknesses in access to health is essential because it will allow directive interventions for this population in the future [66].

As limitations, we based our study on a public data set, and we did not have access to the original data, which may contribute to selection bias, as we do not know if all the individuals with SARS were included. Some epidemiological data were not attributed in the data set for all individuals, reducing the study power for some statistical analyses; in this context, we performed multiple imputations for missing data. Besides that, we needed to exclude essential patient characteristics, such as educational level, chest x-ray, and high-resolution computed tomography of the lungs, due to the number of missing data (>40%). Some important markers were not included in the data set correctly as the medicine types used during the follow-up, drugs used to manage the comorbidities, and characterization of the comorbidities (eg, the individuals were classified as positive for cardiopathies and chronic respiratory diseases, however, we did not have access for the disease definition), laboratorial findings (eg, hemogram), description of the ventilatory support (if any) measures, absence of social characterization (eg, per capita income), and other. Also, it was impossible to determine each disease from the disease spectrum regarding some patients' comorbidities, such as chronic neurological disease and chronic respiratory disease. This observational study can present confounders since the individuals were not randomly assigned. The race was self-declared, and only five races classified as “official” by the Brazilian Institute of Statistics and Geography, were included, which might bias our study. Since the term race is a social construct, it could also bias our analysis.

## CONCLUSIONS

Alongside several clinical symptoms and comorbidities, we associated race with an enhanced risk of death, especially for Black individuals, individuals with multiracial backgrounds, and Indigenous peoples. Although a similar COVID-19 clinical profile was observed among the five races, individuals from minority groups, such as Black and individuals with multiracial backgrounds, frequently experienced symptoms related to a more severe disease, such as dyspnoea and respiratory discomfort, as well as a specific comorbidities profile.

## Additional material


Online Supplementary Document
