# Supplementary material for: Characterization of demographic data, clinical signs, comorbidities, and outcomes according to the race in hospitalized individuals with COVID-19 in Brazil: An observational study
Source: J Glob Health. 2022 Jul 25;12:05027. doi: 10.7189/jogh.12.05027 (PMC9309002; doi:10.7189/jogh.12.05027)
Supplement: Online Supplementary Document [file jogh-12-05027-s001.pdf]

Title: Characterization of Demographic Data, Clinical Signs, Comorbidities, and Outcomes according to the Race in Hospitalized Individuals with COVID-19 in Brazil: An Observational Study

Authors: Nathália Mariana Santos Sansone; Matheus Negri Boschiero; Felipe Eduardo Valencise; Camila Vantini Capasso Palamim; Fernando Augusto Lima Marson

We retrieved the individuals' data from the Brazilian Ministry of Health (https://opendatasus.saude.gov.br/) platform and corresponded to one year of the Coronavirus Pandemic (COVID)-19 pandemic (from February 22, 2020, to April 04, 2021) in Brazil.

Distribution of the individuals infected by the SARS-CoV-2 during the first year of the COVID-19 pandemic in Brazil

Table S1. Epidemiological week of the notification of severe acute respiratory syndrome (SARS) cases due to SARS-CoV-2 infection (Coronavirus Disease (COVID)-19).

Week of notification	Death	Clinical recovery	Total
8	0 (0.0%)	2 (0.0%)	2 (0.0%)
9	0 (0.0%)	4 (0.0%)	4 (0.0%)
10	0 (0.0%)	9 (0.0%)	9 (0.0%)
11	6 (0.0%)	26 (0.0%)	32 (0.0%)
12	165 (0.1%)	294 (0.1%)	459 (0.1%)
13	588 (0.3%)	1,087 (0.3%)	1,675 (0.3%)
14	980 (0.4%)	2,020 (0.6%)	3,000 (0.5%)
15	1,199 (0.5%)	1,951 (0.5%)	3,150 (0.5%)
16	1,788 (0.8%)	2,614 (0.7%)	4,402 (0.8%)
17	2,547 (1.2%)	3,482 (1.0%)	6,029 (1.0%)
18	3,105 (1.4%)	4,607 (1.3%)	7,712 (1.3%)
19	3,944 (1.8%)	5,162 (1.4%)	9,106 (1.6%)
20	4,270 (1.9%)	6,068 (1.7%)	10,338 (1.8%)
21	4,476 (2.0%)	6,838 (1.9%)	11,314 (1.9%)
22	4,252 (1.9%)	6,798 (1.9%)	11,050 (1.9%)
23	4,113 (1.9%)	6,673 (1.8%)	10,786 (1.8%)
24	4,314 (2.0%)	7,047 (1.9%)	11,361 (1.9%)
25	4,546 (2.1%)	8,156 (2.2%)	12,702 (2.2%)
26	4,485 (2.0%)	8,282 (2.3%)	12,766 (2.2%)
27	4,557 (2.1%)	8,513 (2.3%)	13,070 (2.2%)
28	4,883 (2.2%)	9,235 (2.5%)	14,118 (2.4%)
29	4,984 (2.3%)	9,103 (2.5%)	14,087 (2.4%)
30	4,938 (2.3%)	9,417 (2.6%)	14,355 (2.5%)
31	4,771 (2.2%)	8,785 (2.4%)	13,556 (2.3%)
32	4,410 (2.0%)	8,098 (2.2%)	12,508 (2.1%)
33	4,233 (1.9%)	7,874 (2.1%)	12,107 (2.1%)
34	4,038 (1.8%)	7,597 (2.1%)	11,635 (2.0%)
35	3,759 (1.7%)	7,517 (2.1%)	11,276 (1.9%)
36	3,408 (1.6%)	7,069 (1.9%)	10,477 (1.8%)
37	3,185 (1.5%)	6,941 (1.8%)	9,676 (1.7%)
38	2,921 (1.3%)	6,174 (1.7%)	9,095 (1.6%)
39	2,684 1.2%)	5,513 (1.5%)	8,197 (1.4%)
40	2,568 (1.2%)	5,456 (1.5%)	8,024 (1.4%)
41	2,306 (1.1%)	4,827 (1.3%)	7,133 (1.2%)
42	2,043 (0.9%)	4,425 (1.2%)	6,468 (1.1%)
43	1,942 (0.9%)	4,472 (1.3%)	6,414 (1.1%)
44	1,819 (0.8%)	4,498 (1.2%)	6,317 (1.1%)
45	1,992 (0.9%)	4,313 (1.2%)	6,305 (1.1%)
46	2,345 (1.1%)	5,238 (1.4%)	7,593 (1.3%)
47	2,862 (1.3%)	6,697 (1.8%)	9,559 (1.6%)
48	3,486 (1.6%)	7,664 (2.1%)	11,150 (1.9%)
49	3,970 (1.8%)	8,284 (2.3%)	12,254 (2.2%)
50	4,238 (1.9%)	8,444 (2.3%)	12,682 (2.2%)
51	4,104 (1.9%)	8,025 (2.2%)	12,129 (2.1%)
52	3,676 (1.7%)	6,976 (1.9%)	10,652 (1.8%)
53	4,114 (1.9%)	7,574 (2.1%)	11,688 (2.0%)
54	5,256 (2.4%)	9,182 (2.5%)	14,438 (2.5%)
55	5,450 (2.5%)	9,195 (2.5%)	14,645 (2.5%)
56	4,938 (2.3%)	8,519 (2.3%)	13,457 (2.3%)
57	4,647 (2.1%)	7,423 (2.0%)	12,070 (2.1%)

58	4,685 (2.1%)	7,555 (2.1%)	12,240 (2.1%)
59	4,769 (2.2%)	7,511 (2.0%)	12,280 (2.1%)
60	5,367 (2.4%)	8,243 (2.2%)	13,610 (2.3%)
61	7,131 (3.3%)	10,119 (2.8%)	17,250 (2.9%)
62	8,995 (4.1%)	10,812 (2.9%)	19,807 (3.4%)
63	10,011 (4.6%)	10,987 (3.0%)	20,998 (3.6%)
64	9,505 (4.3%)	9,882 (2.7%)	19,387 (3.3%)
65	6,629 (3.0%)	6,058 (1.7%)	12,687 (2.2%)
66	2,696 (1.2%)	1,591 (0.4%)	4,287 (0.7%)
67	46 (0.0%)	31 (0.0%)	77 (0.0%)
Total	219,138 (100%)	366,517 (100%)	585,655 (100%)

SARS-CoV-2, severe acute respiratory syndrome 2.
We obtained the data for the first 67 epidemiological weeks of the COVID-19 pandemic in Brazil (from February 22, 2020, to April 04, 2021). We obtained the data at OpenDataSUS (https://opendatasus.saude.gov.br/).
We presented the data as the number of individuals (N) and the percentage (%).

Table S2. Epidemiological week of the first clinical symptom related to the severe acute respiratory syndrome (SARS) due to SARS-CoV-2 (Coronavirus Disease (COVID)-19) infection.

Week of the first clinical symptom	Death	Clinical recovery	Total
8	12 (0.0%)	20 (0.0%)	32 (0.0%)
9	11 (0.0%)	2 (0.0%)	37 (0.0%)
10	48 (0.0%)	87 (0.0%)	135 (0.0%)
11	174 (0.1%)	385 (0.1%)	559 (0.1%)
12	612 (0.3%)	1,263 (0.3%)	1875 (0.3%)
13	952 (0.4%)	1,851 (0.5%)	2,803 (0.5%)
14	1,422 (0.6%)	2,166 (0.6%)	3,588 (0.6%)
15	1,940 (0.9%)	2,794 (0.8%)	4,734 (0.8%)
16	2,727 (1.2%)	3,824 (1.0%)	6,551 (1.1%)
17	35,543 (1.6%)	4,986 (1.4%)	8,529 (1.5%)
18	4,552 (2.1%)	5,840 (1.6%)	10,392 (1.8%)
19	4,630 (2.1%)	6,307 (1.7%)	10,937 (1.9%)
20	4,961 (2.3%)	7,396 (2.0%)	12,357 (2.1%)
21	4,369 (2.0%)	7,001 (1.9%)	11,370 (1.9%)
22	4,016 (1.8%)	6,584 (1.8%)	10,600 (1.8%)
23	4,555 (2.1%)	7,824 (2.1%)	12,379 (2.1%)
24	4,282 (2.0%)	8,120 (2.2%)	12,402 (2.1%)
25	4,625 (2.1%)	9,005 (2.5%)	13,630 (2.3%)
26	4,235 (1.9%)	8,105 (2.2%)	12,340 (2.1%)
27	5,048 (2.3%)	9,440 (2.6%)	14,488 (2.5%)
28	5,231 (2.4%)	9,474 (2.6%)	14,704 (2.5%)
29	4,771 (2.2%)	8,897 (2.4%)	13,668 (2.3%)
30	4,773 (2.2%)	8,645 (2.4%)	13,418 (2.3%)
31	4,411 (2.0%)	8,173 (2.2%)	12,584 (2.1%)
32	3,932 (1.8%)	7,523 (2.1%)	11,455 (2.0%)
33	4,013 (1.8%)	7,722 (2.1%)	11,735 (2.0%)
34	3,527 (1.6%)	7,247 (2.0%)	10,774 (1.8%)
35	3,134 (1.4%)	6,573 (1.8%)	9,707 (1.7%)
36	3,210 (1.5%)	6,479 (1.8%)	9,689 (1.7%)
37	2,796 (1.3%)	5,892 (1.6%)	8,688 (1.5%)
38	2,534 (1.2%)	5,296 (1.4%)	7,830 (1.3%)
39	2,353 (1.1%)	5,160 (1.4%)	7,513 (1.3%)
40	2,322 (1.1%)	4,766 (1.3%)	7,088 (1.2%)
41	2,075 (0.9%)	4,597 (1.2%)	6,672 (1.1%)
42	1,734 (0.8%)	4,244 (1.2%)	5,978 (1.0%)
43	1,879 (0.9%)	4,364 (1.2%)	6,243 (1.1%)
44	1,872 (0.9%)	4,383 (1.2%)	6,255 (1.1%)
45	2,448 (1.1%)	5,918 (1.6%)	8,366 (1.4%)
46	2,989 (1.4%)	6,939 (1.9%)	9,928 (1.7%)
47	3,694 (1.7%)	8,102 (2.2%)	11,796 (2.0%)
48	3,871 (1.8%)	8,165 (2.2%)	12,036 (2.0%)
49	4,596 (2.1%)	8,691 (2.4%)	13,287 (2.3%)
50	4,229 (1.9%)	7,859 (2.1%)	12,088 (2.1%)
51	3,955 (1.8%)	7,265 (2.0%)	11,220 (1.9%)
52	4,501 (2.1%)	8,092 (2.2%)	12,593 (2.2%)
53	5,395 (2.5%)	9,152 (2.5%)	14,547 (2.5%)
54	5,437 (2.5%)	9,145 (2.5%)	14,582 (2.5%)
55	5,254 (2.4%)	8,300 (2.3%)	13,554 (2.4%)
56	4,562 (2.1%)	7,514 (2.1%)	12,076 (2.1%)
57	4,067 (1.9%)	6,915 (1.9%)	10,982 (1.9%)
58	5,114 (2.3%)	7,975 (2.2%)	13,089 (2.2%)
59	5,797 (2.6%)	8,848 (2.4%)	14,645 (2.5%)
60	7,932 (3.6%)	10,611 (2.9%)	18,543 (3.2%)
61	8,602 (3.9%)	10,381 (2.8%)	18,983 (3.2%)

62	10,878 (5.0%)	11,586 (3.2%)	22,464 (3.8%)
63	8,418 (3.8%)	8,132 (2.2%)	16,550 (2.8%)
64	4,774 (2.2%)	3,734 (1.0%)	8,508 (1.5%)
65	1,237 (0.6%)	696 (0.2%)	1,933 (0.35)
66	107 (0.0%)	39 (0.0%)	146 (0.0%)
Total	219,138 (100%)	366,517 (100%)	585,655 (100%)

SARS-CoV-2, severe acute respiratory syndrome 2.
We obtained the data for the first 67 epidemiological weeks of the COVID-19 pandemic in Brazil (from February 22, 2020, to April 04, 2021). We obtained the data at OpenDataSUS (https://opendatasus.saude.gov.br/).
We presented the data as the number of individuals (N) and the percentage (%).

Table S3. Number and percentage of individuals with Coronavirus Disease (COVID)-19 in the Brazilian states and Federal District.

**State**	**N**	**%**
Acre	627	0.1
Alagoas	4,504	0.8
Amazonas	20,843	3.6
Amapá	2,687	0.5
Bahia	19,656	3.4
Ceará	16,680	2.8
Federal District	8,560	1.5
Espírito Santo	3,387	0.6
Goiás	20,116	3.4
Maranhão	4,349	0.7
Minas Gerais	58,797	10.0
Mato Grosso do Sul	10,769	1.8
Mato Grosso	12,133	2.1
Pará	18,201	3.1
Paraíba	10,776	1.8
Pernambuco	8,360	1.4
Piauí	6,495	1.1
Paraná	38,289	6.5
Rio de Janeiro	37,082	6.3
Rio Grande do Norte	6,196	1.1
Rondônia	5,322	0.9
Roraima	1,700	0.3
Rio Grande do Sul	50,404	8.6
Santa Catarina	27,833	4.8
Sergipe	3,434	0.6
São Paulo	185,380	31.7
Tocantins	3,075	0.5
Total	585,655	100

We presented the data as the number of individuals (N) and the percentage (%).

Association of the outcome and the patients' characteristics with the SARS-CoV-2 infection during the first year of the COVID-19 pandemic in Brazil – All individuals

Table S4. Association between the case fatality rate due to SARS-CoV-2 infection in hospitalized Brazilian individuals' regarding to the demographic data.

**Data**	**Category**	**Death**	**Clinical recovery**	**Total**	***P***	**OR**	**95%CI**
Sex	Male	123,726 (56.2%)	200,484 (54.7%)	324,210 (55.4%)	<0.001	1.074	1.063-1.085
	Female	95,412 (43.5%)	166,033 (45.3%)	261,445 (44.6%)		1	Reference
Age	<1-year-old	328 (0.1%)	2,505 (0.7%)	2,833 (0.5%)	-	1	Reference
	1 to 12 years old	285 (0.1%)	5,091 (1.4%)	5,376 (0.9%)	<0.001	0.428	0.362-0.505
	13 to 24 years old	1,256 (0.6%)	9,391 (2.6%)	10,647 (1.8%)	0.748	1.021	0.898-1.162
	25 to 60 years old	58,192 (26.6%)	203,331 (55.5%)	261,523 (44.7%)	<0.001	2.186	1.947-2.453
	61 to 72 years old	65,072 (29.7%)	83,437 (22.8%)	148,509 (25.4%)	<0.001	5.956	5.306-6.686
	73 to 85 years old	68,125 (31.1%)	50,532 (13.8%)	118,657 (20.3%)	<0.001	10.300	9.172-11.560
	+85 years old	25,880 (11.8%)	12,230 (3.3%)	38,110 (6.5%)	<0.001	16.160	14.380-18.170
Place of residence	Urban	207,431 (94.7%)	350,140 (95.5%)	557,571 (95.2%)	-	1	Reference
	Rural	10,941 (5.0%)	15,274 (4.2%)	26,215 (4.5%)	<0.001	1.209	1.179-1.240
	Peri-urban	766 (0.3%)	1,103 (0.3%)	1,869 (0.3%)	<0.001	1.172	1.069-1.286
Lived in a place with a Flu outbreak	Yes	47,075 (21.5%)	74,004 (20.2%)	121,079 (20.7%)	<0.001	1.081	1.067-1.096
	No	172,063 (78.5%)	292,513 (79.8%)	464,576 (79.3%)		1	Reference
Nosocomial infection	Yes	6,339 (2.9%)	4,952 (1.4%)	11,291 (1.9%)	<0.001	2.175	2.095-2.258
	No	212,799 (97.1%)	361,565 (98.6%)	574,364 (98.1%)		1	Reference

SARS-CoV-2, severe acute respiratory syndrome 2; OR, odds ratio; 95%CI, 95% confidence interval.
We presented the data as the number of individuals (N) and the percentage (%).
We performed the statistical analysis using the χ^2 test. Alpha=0.05.

Table S5. Association between the case fatality rate due to SARS-CoV-2 infection in hospitalized Brazilian individuals' regarding to the clinical symptoms.

Data	Category	Death	Clinical recovery	Total	*P*	OR	95%CI
Fever	Yes	151,047 (68.9%)	261,036 (71.2%)	412,083 (70.4%)	<0.001	0.896	0.886-0.907
	No	68,091 (31.1%)	105,481 (28.8%	173,572 (29.6%)		1	Reference
Cough	Yes	169,018 (77.1%)	294,458 (80.3%)	463,476 (79.1%)	<0.001	0.825	0.882-0.896
	No	50,120 (22.9%)	72,059 (19.7%)	122,179 (20.9%)		1	Reference
Sore throat	Yes	61,844 (28.2%)	116,774 (31.9%)	178,618 (30.5%)	<0.001	0.841	0.831-0.851
	No	157,294 (71.8%)	249,743 (68.1%)	407,037 (69.5%)		1	Reference
Dyspnea	Yes	191,239 (87.3%)	280,720 (76.6%)	471,959 (80.6%)	<0.001	2.095	2.064-2.126
	No	27,899 (12.7%)	85,797 (23.4%)	113,696 (19.4%)		1	Reference
Respiratory discomfort	Yes	177,402 (81.0%)	250,292 (68.3%)	427,694 (73.0%)	<0.001	1.974	1.949-1.999
	No	41,736 (19.0%)	116,225 (31.7%)	157,961 (27.0%)		1	Reference
Oxygen saturation	<95%	184,813 (84.3%)	242,339 (66.1%)	427,152 (72.9%)	<0.001	2.759	2.722-2.796
	≥95%	34,325 (15.7%)	124,178 (33.9%)	158,503 (27.1%)		1	Reference
Diarrhea	Yes	55,163 (25.2%)	100,222 (27.3%)	155,385 (26.5%)	<0.001	0.894	0.883-0.905
	No	163,975 (74.8%)	266,295 (72.7%)	430,270 (73.5%)		1	Reference
Vomit	Yes	42,717 (19.5%)	75,553 (20.6%)	118,270 (20.2%)	<0.001	0.933	0.920-0.945
	No	176,421 (80.5%)	290,964 (79.4%)	467,385 (79.8%)		1	Reference
Abdominal pain	Yes	39,736 (18.1%)	65,162 (17.8%)	104,898 (17.9%)	0.001	1.024	1.010-1.039
	No	179,402 (81.9%)	301,355 (82.2%)	470,757 (82.1%)		1	Reference
Fatigue	Yes	82,335 (37.6%)	130,578 (35.6%)	212,913 (36.4%)	<0.001	1.087	1.076-1.099
	No	136,803 (62.4%)	235,939 (64.4%)	372,742 (63.6%)		1	Reference
Loss of smell	Yes	49,018 (22.4%)	95,545 (26.1%)	144,563 (24.7%)	<0.001	0.817	0.807-0.827
	No	170,120 (77.6%)	270,972 (73.9%)	441,092 (76.3%)		1	Reference
Loss of taste	Yes	48,240 (22.5%)	96,988 (26.5%)	146,228 (25.0%)	<0.001	0.805	0.796-0.816
	No	169,898 (77.5%)	269,529 (73.5%)	439,427 (75.0%)		1	Reference
Other symptoms	Yes	90,358 (41.2%)	174,492 (47.6%)	264,850 (45.2%)	<0.001	0.772	0.764-0.780
	No	128,780 (58.8%)	192,025 (52.4%)	320,805 (54.8%)		1	Reference

SARS-CoV-2, severe acute respiratory syndrome 2; OR, odds ratio; 95%CI, 95% confidence interval.
We presented the data as the number of individuals (N) and the percentage (%).
We performed the statistical data analysis using the χ^2 test. Alpha=0.05.

Table S6. Association between the case fatality rate due to SARS-CoV-2 infection in hospitalized Brazilian individuals' regarding to the comorbidities.

**Data**	**Category**	**Death**	**Clinical recovery**	**Total**	***P***	OR	**95%CI**
Comorbidities (any)	Yes	171,508 (78.3%)	218,235 (59.5%)	389,743 (66.5%)	<0.001	2.447	2.417-2.476
	No	47,630 (21.7%)	148,282 (40.5%)	195,912 (33.5%)		1	Reference
Cardiopathy	Yes	123,420 (56.3%)	139,945 (38.2%)	263,365 (45.0%)	<0.001	2.088	2.065-2.110
	No	95,718 (43.7%)	226,572 (61.8%)	322,290 (55.0%)		1	Reference
Hematologic disorder	Yes	2,883 (1.3%)	3,173 (0.9%)	6,056 (1.0%)	<0.001	1.527	1.451-1.606
	No	216,255 (98.7%)	363,344 (99.1%)	579,599 (99.0%)		1	Reference
Down syndrome	Yes	782 (0.4%)	950 (0.3%)	1,732 (0.3%)	<0.001	1.378	1.253-1.515
	No	218,356 (99.6%)	365,567 (99.7%)	583,923 (99.7%)		1	Reference
Hepatic disorder	Yes	3,947 (1.8%)	4,585 (1.3%)	8,532 (1.5%)	<0.001	1.448	1.387-1.511
	No	215,191 (98.2%)	361,932 (98.7%)	577,123 (98.5%)		1	Reference
Asthma	Yes	15,215 (6.9%)	31,119 (8.5%)	46,334 (7.9%)	<0.001	0.804	0.788-0.821
	No	203,923 (93.1%)	335,398 (91.5%)	539,321 (92.1%)		1	Reference
Diabetes mellitus	Yes	105,109 (48.0%)	113,649 (31.0%)	218,758 (37.4%)	<0.001	2.051	2.029-2.073
	No	114,029 (52.0%)	252,868 (69.0%)	366,897 (62.6%)		1	Reference
Neurological disorder	Yes	27,045 (12.3%)	33,609 (9.2%)	60,654 (10.4%)	<0.001	1.395	1.371-1.418
	No	192,093 (87.7%)	332,908 (90.8%)	525,001 (89.6%)		1	Reference
Chronic respiratory disease	Yes	28,220 (12.9%)	37,073 (10.1%)	65,293 (11.1%)	<0.001	1.314	1.292-1.335
	No	190,918 (87.1%)	329,444 (89.9%)	520,362 (88.9%)		1	Reference
Immunosuppressive disorder	Yes	18,145 (8.3%)	27,507 (7.5%)	45,652 (7.8%)	<0.001	1.113	1.091-1.135
	No	200,993 (91.7%)	339,010 (92.5%)	540,003 (92.2%)		1	Reference
Renal disease	Yes	29,584 (13.5%)	38,083 (10.4%)	67,667 (11.6%)	<0.001	1.346	1.324-1.368
	No	189,554 (86.5%)	328,434 (89.6%)	517,988 (88.4%)		1	Reference
Obesity	Yes	47,373 (21.6%)	63,846 (17.4%)	111,219 (19.0%)	<0.001	1.307	1.290-1.325
	No	171,765 (78.4%)	302,671 (82.6%)	474,436 (81.0%)		1	Reference
Other comorbidities	Yes	112,579 (51.4%)	124,260 (33.9%)	236,839 (40.4%)	<0.001	2.060	2.038-2.082
	No	106,559 (48.6%)	242,257 (66.1%)	348,816 (59.6%)		1	Reference

SARS-CoV-2, severe acute respiratory syndrome 2; OR, odds ratio; 95%CI, 95% confidence interval.
We presented the data as the number of individuals (N) and the percentage (%).
We performed the statistical analysis using the χ^2 test. Alpha=0.05.

Table S7. Association between the case fatality rate due to SARS-CoV-2 infection in hospitalized Brazilian individuals' regarding to the antiviral drugs used to treat the infection and the hospitalization data.

Data	Category	Death	Clinical recovery	Total	*P*	OR	95%CI
Antiviral drugs to treat the infection	Yes	27,665 (12.6%)	50,488 (13.8%)	78,153 (13.3%)	<0.001	0.904	0.890-0.919
	No	191,473 (87.4%)	316,029 (86.2%)	507,502 (86.7%)		1	Reference
Need for intensive care unit	Yes	131,870 (60.2%)	80,197 (21.9%)	212,067 (36.2%)	<0.001	5.395	5.333-5.458
	No	87,268 (39.8%)	286,320 (78.1%)	373,588 (63.8%)		1	Reference
Need for ventilatory support	Invasive	103,478 (47.2%)	23,041 (6.3%)	126,519 (21.6%)	<0.001	22.850	22.400-23.310
	Non-invasive	82,484 (42.2%)	225,548 (61.5%)	318,032 (54.3%)		2.086	2.053-2.120
	None required	23,176 (10.6%)	117,928 (32.2%)	141,104 (24.1%)		1	Reference
Discharge criterion	Laboratorial criterion	204,029 (93.1%)	344,571 (94.0%)	548,600 (93.7%)	<0.001	1	Reference
	Clinical criterion	15,109 (6.9%)	21,946 (6.0%)	37,055 (6.3%)		1.163	1.138-1.188

SARS-CoV-2, severe acute respiratory syndrome 2; OR, odds ratio; 95%CI, 95% confidence interval.
We presented the data as the number of individuals (N) and the percentage (%).
We performed the statistical analysis using the χ^2 test. Alpha=0.05.

Association of the outcome and the patients' characteristics with the SARS-CoV-2 infection during the first year of the COVID-19 pandemic in Brazil – White individuals

Table S8. Association between the case fatality rate due to SARS-CoV-2 infection in hospitalized Brazilian individuals' regarding to the demographic data.

Data	Category	Death	Clinical recovery	Total	*P*	OR	95%CI
Sex	Male	61,438 (55.6%)	108,097 (54.3%)	169,535 (54.8%)	<0.001	1.055	1.039-1.071
	Female	49,057 (44.4%)	91,054 (45.7%)	140,111 (45.2%)		1	Reference
Age	<1-year-old	88 (0.1%)	956 (0.5%)	1,044 (0.3%)		1	Reference
	1 to 12 years old	89 (0.1%)	1,714 (0.9%)	1,803 (0.6%)	<0.001	0.564	0.416-0.765
	13 to 24 years old	452 (0.4%)	3,940 (2.0%)	4,392 (1.4%)	0.080	1.246	0.981-1.583
	25 to 60 years old	25,939 (23.5%)	108,560 (54.5%)	134,499 (43.4%)	<0.001	2.596	2.086-3.230
	61 to 72 years old	32,652 (29.6%)	47,211 (23.7%)	79,862 (25.8%)	<0.001	7.513	6.037-9.351
	73 to 85 years old	36,352 (32.9%)	29,340 (14.7%)	65,682 (21.2%)	<0.001	13.510	10.850-16.820
	+85 years old	14,823 (13.5%)	7,430 (3.7%)	22,353 (7.2%)	<0.001	21.820	17.510-27.190
Place of residence	Urban	106,178 (96.1%)	191,967 (96.4%)	298,145 (96.3%)		1	Reference
	Rural	3,988 (3.6%)	6,603 (3.3%)	10,591 (3.4%)	<0.001	1.092	1.049-1.137
	Peri-urban	329 (0.3%)	581 (0.3%)	910 (0.3%)	0.760	1.024	0.894-1.172
Lived in a place with a Flu outbreak	Yes	18,676 (16.9%)	33,710 (16.7%)	52,386 (16.9%)	0.860	0.998	0.979-1.018
	No	91,819 (83.1%)	16,5441 (83.1%)	257,260 (83.1%)		1	Reference
Nosocomial infection	Yes	3,390 (3.1%)	2,512 (1.3%)	5,902 (1.9%)	<0.001	2.478	2.352-2.610
	No	107,105 (96.9%)	196,639 (98.7%)	303,744 (98.1%)		1	Reference

SARS-CoV-2, severe acute respiratory syndrome 2; OR, odds ratio; 95%CI, 95% confidence interval.
We presented the data as the number of individuals (N) and the percentage (%).
We performed the statistical analysis using the χ^2 test. Alpha=0.05.

Table S9. Association between the case fatality rate due to SARS-CoV-2 infection in hospitalized Brazilian individuals' regarding to the clinical symptoms.

**Data**	**Category**	**Death**	**Clinical recovery**	**Total**	***P***	**OR**	**95%CI**
Fever	Yes	73,269 (66.3%)	138,318 (69.5%)	211,587 (68.3%)	<0.001	0.866	0.852-0.879
	No	37,226 (33.7%)	60,833 (30.5%)	98,056 (31.7%)		1	Reference
Cough	Yes	83,642 (75.7%)	158,449 (79.6%)	242,091 (78.2%)	<0.001	0.800	0.786-0.814
	No	26,853 (24.3%)	40,702 (20.4%)	67,555 (21.8%)		1	Reference
Sore throat	Yes	30,366 (27.5%)	62,576 (31.4%)	92,942 (30.0%)	<0.001	0.827	0.814-0.841
	No	80,129 (72.5%)	136,575 (68.6%)	216,704 (70.0%)		1	Reference
Dyspnea	Yes	95,636 (86.6%)	151,969 (76.3%)	247,605 (80.0%)	<0.001	1.998	1.958-2.039
	No	14,859 (13.4%)	47,182 (23.7%)	62,041 (20.0%)		1	Reference
Respiratory discomfort	Yes	88,718 (80.3%)	135,500 (68.0%)	224,218 (72.4%)	<0.001	1.914	1.880-1.948
	No	21,777 (19.7%)	63,651 (32.0%)	85,428 (27.6%)		1	Reference
Oxygen saturation	<95%	94,077 (85.1%)	135,108 (67.8%)	229,185 (74.0%)	<0.001	2.716	2.665-2.768
	≥95%	16,418 (14.9%)	64,043 (32.2%)	80,461 (26.0%)		1	Reference
Diarrhea	Yes	28,802 (26.1%)	56,142 (28.2%)	84,944 (27.4%)	<0.001	0.898	0.883-0.913
	No	81,693 (73.9%)	143,009 (71.8%)	224,702 (72.6%)		1	Reference
Vomit	Yes	22,135 (20.0%)	41,335 (20.8%)	63,470 (20.5%)	<0.001	0.956	0.939-0.974
	No	88,360 (80.0%)	157,816 (79.2%)	246,176 (79.5%)		1	Reference
Abdominal pain	Yes	20,676 (18.7%)	36,322 (18.2%)	56,998 (18.4%)	0.001	1.032	1.013-1.052
	No	89,819 (81.3%)	162,829 (81.8%)	252,648 (81.6%)		1	Reference
Fatigue	Yes	44,657 (40.4%)	77,321 (38.8%)	121,978 (39.4%)	<0.001	1.069	1.053-1.085
	No	65,838 (59.6%)	121,830 (61.2%)	187,668 (60.6%)		1	Reference
Loss of smell	Yes	24,929 (22.6%)	52,082 (26.2%)	77,011 (24.9%)	<0.001	0.823	0.809-0.837
	No	85,566 (77.4%)	147,069 (73.8%)	232,635 (75.1%)		1	Reference
Loss of taste	Yes	25,152 (22.8%)	53,228 (26.7%)	78,380 (25.3%)	<0.001	0.808	0.794-0.822
	No	85,343 (77.2%)	145,923 (73.3%)	231,266 (74.7%)		1	Reference
Other symptoms	Yes	46,782 (42.3%)	96,948 (48.7%)	143,730 (46.4%)	<0.001	0.774	0.763-0.786
	No	63,713 (57.7%)	102,203 (51.3%)	165,916 (53.6%)		1	Reference

SARS-CoV-2, severe acute respiratory syndrome 2; OR, odds ratio; 95%CI, 95% confidence interval.
We presented the data as the number of individuals (N) and the percentage (%).
We performed the statistical data analysis using the χ^2 test. Alpha=0.05.

Table S10. Association between the case fatality rate due to SARS-CoV-2 infection in hospitalized Brazilian individuals' regarding to the comorbidities. – White patients

**Data**	**Category**	**Death**	**Clinical recovery**	**Total**	***P***	OR	**95%CI**
Comorbidities (any)	Yes	90,266 (81.7%)	121,720 (61.1%)	211,986 (68.5%)	<0.001	2.839	2.789-2.889
	No	20,229 (18.3%)	77,431 (38.9%)	97,660 (31.5%)		1	Reference
Cardiopathy	Yes	65,673 (59.4%)	79,060 (39.7%)	144,733 (46.7%)	<0.001	2.226	2.192-2.259
	No	44,822 (40.6%)	120,091 (60.3%)	164,913 (53.3%)		1	Reference
Hematologic disorder	Yes	1,605 (1.5%)	1,719 (0.9%)	3,324 (1.1%)	<0.001	1.693	1.581-1.813
	No	108,890 (98.5%)	197,432 (99.1%)	306,322 (98.9%)		1	Reference
Down syndrome	Yes	398 (0.4%)	559 (0.3%)	957 (0.3%)	<0.001	1.284	1.129-1.461
	No	110,097 (99.6%)	198,592 (99.7%)	308,689 (99.7%)		1	Reference
Hepatic disorder	Yes	2,093 (1.9%)	2,612 (1.3%)	4,705 (1.5%)	<0.001	1.453	1.371-1.540
	No	108,402 (98.1%)	196,539 (98.7%)	304,941 (98.5%)		1	Reference
Asthma	Yes	8,579 (7.8%)	1,7685 (8.9%)	26,264 (8.5%)	<0.001	0.864	0.841-0.887
	No	101,916 (92.2%)	181,466 (91.1%)	283,382 (91.5%)		1	Reference
Diabetes mellitus	Yes	5,4102 (49.0%)	62,504 (31.4%)	116,606 (37.7%)	<0.001	2.097	2.066-2.129
	No	56,393 (51.0%)	136,647 (68.6%)	193,040 (62.3%)		1	Reference
Neurological disorder	Yes	15,915 (14.4%)	19,409 (9.7%)	35,324 (11.4%)	<0.001	1.558	1.524-1.596
	No	94,580 (85.6%)	179,742 (90.3%)	274,322 (88.6%)		1	Reference
Chronic respiratory disease	Yes	15,998 (14.5%)	21,109 (10.6%)	37,107 (12.0%)	<0.001	1.428	1.397-1.460
	No	94,497 (85.5%)	178,042 (89.4%)	272,539 (88.0%)		1	Reference
Immunosuppressive disorder	Yes	10,150 (9.2%)	15,472 (7.8%)	25,622 (8.3%)	<0.001	1.201	1.170-1.233
	No	100,345 (90.8%)	183,679 (92.2%)	284,024 (91.7%)		1	Reference
Renal disease	Yes	15,647 (14.2%)	20,950 (10.5%)	36,597 (11.8%)	<0.001	1.403	1.372-1.435
	No	94,848 (95.5%)	178,201 (89.5%)	273,049 (88.2%)		1	Reference
Obesity	Yes	25,711 (23.3%)	36,881 (18.5%)	62,592 (20.2%)	<0.001	1.334	1.311-1.358
	No	84,784 (76.7%)	162,270 (81.5%)	247,054 (79.8%)		1	Reference
Other comorbidities	Yes	58,947 (53.3%)	688,23 (34.6%)	127,770 (41.3%)	<0.001	2.165	2.133-2.198
	No	51,548 (46.7%)	130,328 (65.4%)	181,876 (58.7%)		1	Reference

SARS-CoV-2, severe acute respiratory syndrome 2; OR, odds ratio; 95%CI, 95% confidence interval.

We presented the data as the number of individuals (N) and the percentage (%).

We performed the statistical analysis using the χ^2 test. Alpha=0.05.

Table S11. Association between the case fatality rate due to SARS-CoV-2 infection in hospitalized Brazilian individuals' regarding to the antiviral drugs used to treat the infection and the hospitalization data.

Data	Category	Death	Clinical recovery	Total	*P*	OR	95%CI
Antiviral drugs to treat the infection	Yes	13,750 (12.4%)	28,718 (14.4%)	42,468 (13.7%)	<0.001	0.844	0.825-0.862
	No	96,745 (87.6%)	170,433 (85.6%)	267,178 (86.3%)		1	Reference
Need for intensive care unit	Yes	68,366 (61.9%)	45,228 (22.7%)	113,594 (36.7%)	<0.001	5.523	5.235-5.612
	No	42,129 (38.1%)	153,923 (77.3%)	196,052 (63.3%)		1	Reference
Need for ventilatory support	Invasive	51,520 (46.6%)	12,979 (6.5%)	64,499 (20.8%)	<0.001	23.590	22.930-24.270
	Non-invasive	48,656 (44.0%	124,851 (62.7%)	173,507 (56.0%)	<0.001	2.316	2.262-2.371
	None required	10,319 (9.3%)	61,321 (30.8%)	71,640 (23.1%)		1	Reference
Discharge criterion	Laboratorial criterion	104,705 (94.8%)	190,075 (95.4%)	294,780 (95.2%)	<0.001	1	Reference
	Clinical criterion	5,790 (5.2%)	90,76 (4.6%)	14,866 (4.8%)		1.158	1.120-1.198

SARS-CoV-2, severe acute respiratory syndrome 2; OR, odds ratio; 95%CI, 95% confidence interval.
We presented the data as the number of individuals (N) and the percentage (%).
We performed the statistical analysis using the χ^2 test. Alpha=0.05.

Association of the outcome and the patients' characteristics with the SARS-CoV-2 infection during the first year of the COVID-19 pandemic in Brazil – Black individuals.

Table S12. Association between the case fatality rate due to SARS-CoV-2 infection in hospitalized Brazilian individuals' regarding to the demographic data.

Data	Category	Death	Clinical recovery	Total	*P*	OR	95%CI
Sex	Male	7,464 (56.2%)	10,170 (54.7%)	17,634 (55.3%)	0.011	1.060	1.014-1.109
	Female	5,825 (43.8%)	8,413 (45.3%)	14,238 (44.7%)		1	Reference
Age	<1-year-old	9 (0.1%)	82 (0.4%)	91 (0.3%)		1	Reference
	1 to 12 years old	17 (0.1%)	193 (1.0%)	210 (0.7%)	0.775	0.803	0.344-1.874
	13 to 24 years old	79 (0.6%)	470 (2.5%)	549 (1.7%)	0.322	1.531	0.739-3.172
	25 to 60 years old	4,018 (30.2%)	10,815 (58.2%)	14,833 (46.5%)	<0.001	3.385	1.699-6.743
	61 to 72 years old	4,155 (31.3%)	4,208 (22.6%)	8,363 (26.2%)	<0.001	8.996	4.514-17.930
	73 to 85 years old	3,756 (28.3%)	2,326 (12.5%)	6,082 (19.1%)	<0.001	14.710	7.378-29.340
	+85 years old	1,255 (9.4%)	489 (2.6%)	1,744 (5.5%)	<0.001	23.380	11.660-46.900
Place of residence	Urban	12,717 (95.7%)	17,922 (96.4%)	30,639 (96.1%)		1	Reference
	Rural	521 (3.9%)	592 (3.2%)	1,113 (3.5%)	<0.001	1.240	1.100-1.398
	Peri-urban	51 (0.4%)	69 (0.4%)	120 (0.4%)	0.898	1.042	0.725-1.497
Lived in a place with a Flu outbreak	Yes	2,957 (22.3%)	3,789 (20.4%)	6,746 (21.2%)	<0.001	1.117	1.058-1.180
	No	10,332 (77.7%)	14,794 (79.6%)	25,126 (78.8%)		1	Reference
Nosocomial infection	Yes	505 (3.8%)	358 (1.9%)	863 (2.7%)	<0.001	2.011	1.753-2.307
	No	12,784 (96.2%)	18,225 (98.1%)	31,009 (97.3%)		1	Reference

SARS-CoV-2, severe acute respiratory syndrome 2; OR, odds ratio; 95%CI, 95% confidence interval.
We presented the data as the number of individuals (N) and the percentage (%).
We performed the statistical analysis using the χ^2 test. Alpha=0.05.

Table S13. Association between the case fatality rate due to SARS-CoV-2 infection in hospitalized Brazilian individuals' regarding to the clinical symptoms.

Data	Category	Death	Clinical recovery	Total	*P*	OR	95%CI
Fever	Yes	9,194 (69.2%)	13,249 (71.3%)	22,443 (70.4%)	<0.001	0.904	0.861-0.949
	No	4,095 (30.8%)	5,334 (28.7%)	9,429 (29.6%)		1	Reference
Cough	Yes	10,379 (78.1%)	15,148 (81.5%)	25,527 (80.1%)	<0.001	0.809	0.765-0.855
	No	2,910 (21.9%)	3,435 (18.5%)	6,345 (19.9%)		1	Reference
Sore throat	Yes	3,736 (28.1%)	5,905 (31.8%)	9,641 (30.2%)	<0.001	0.840	0.800-0.882
	No	9,553 (71.9%)	12,678 (68.2%)	22,231 (69.8%)		1	Reference
Dyspnea	Yes	11,523 (86.7%)	14,322 (77.1%)	25,845 (81.1%)	<0.001	1.941	1.827-2.063
	No	1,766 (13.3%)	4,261 (22.9%)	6,027 (18.9%)		1	Reference
Respiratory discomfort	Yes	10,750 (80.9%)	12,846 (69.1%)	23,596 (74.0%)	<0.001	1.891	1.793-1.994
	No	2,539 (19.1%)	5,737 (30.9%)	8,276 (26.0%)		1	Reference
Oxygen saturation	<95%	11,327 (85.2%)	12,659 (68.1%)	23,986 (75.3%)	<0.001	2.702	2.552-2.860
	≥95%	1,962 (14.8%)	5,924 (31.9%)	7,886 (24.7%)		1	Reference
Diarrhea	Yes	3,670 (27.6%)	5,282 (28.4%)	8,952 (28.1%)	0.114	0.961	0.914-1.010
	No	9,619 (72.4%)	13,301 (71.6%)	22,920 (71.9%)		1	Reference
Vomit	Yes	2,959 (22.3%)	4,204 (22.6%)	7,163 (22.5%)	0.452	0.980	0.929-1.033
	No	10,330 (77.7%)	14,379 (77.4%)	24,709 (77.5%)		1	Reference
Abdominal pain	Yes	2,708 (20.4%)	3,556 (19.1%)	6,264 (19.7%)	0.006	1.082	1.023-1.144
	No	1,0581 (79.6%)	15,027 (80.9%)	25,608 (80.3%)		1	Reference
Fatigue	Yes	5,041 (37.9%)	6,543 (35.2%)	11,584 (36.3%)	<0.001	1.125	1.074-1.178
	No	8,248 (62.1%)	12,040 (64.8%)	20,288 (63.7%)		1	Reference
Loss of smell	Yes	3,255 (24.5%)	5,016 (27.0%)	8,271 (26.0%)	<0.001	0.877	0.834-0.923
	No	10,034 (75.5%)	13,567 (73.0%)	23,601 (74.0%)		1	Reference
Loss of taste	Yes	3,279 (24.7%)	5,054 (27.2%)	8,333 (26.1%)	<0.001	0.877	0.833-0.923
	No	10,010 (75.3%)	13,529 (72.8%)	23,539 (73.9%)		1	Reference
Other symptoms	Yes	5,694 (42.8%)	8,623 (46.4%)	14,317 (44.9%)	<0.001	0.866	0.828-0.906
	No	9,960 (53.6%)	7,595 (57.2%)	17,555 (55.1%)		1	Reference

SARS-CoV-2, severe acute respiratory syndrome 2; OR, odds ratio; 95%CI, 95% confidence interval.

We presented the data as the number of individuals (N) and the percentage (%).

We performed the statistical data analysis using the χ^2 test. Alpha=0.05.

Table S14. Association between the case fatality rate due to SARS-CoV-2 infection in hospitalized Brazilian individuals' regarding to the comorbidities.

Data	Category	Death	Clinical recovery	Total	*P*	OR	95%CI
Comorbidities (any)	Yes	10,806 (81.3%)	12,212 (65.7%)	23,018 (72.2%)	<0.001	2.270	2.153-2.394
	No	2,483 (18.7%)	6,371 (34.3%)	8,854 (27.8%)		1	Reference
Cardiopathy	Yes	8,091 (60.9%)	8,123 (43.7%)	16,214 (50.9%)	<0.001	2.004	1.916-2.097
	No	5,198 (39.1%)	10,460 (56.3%)	15,658 (49.1%)		1	Reference
Hematologic disorder	Yes	195 (1.5%)	224 (1.2%)	419 (1.3%)	0.043	1.221	1.006-1.481
	No	13,094 (98.5%)	18,359 (98.8%)	31,453 (98.7%)		1	Reference
Down syndrome	Yes	48 (0.4%)	47 (0.3%)	95 (0.3%)	0.080	1.430	0.956-2.139
	No	13,241 (99.6%)	18,536 (99.7%)	31,777 (99.7%)		1	Reference
Hepatic disorder	Yes	283 (2.1%)	262 (1.4%)	545 (1.7%)	<0.001	1.522	1.284-1.803
	No	13,006 (97.9%)	18,321 (98.6%)	31,327 (98.3%)		1	Reference
Asthma	Yes	1,083 (8.1%)	1,784 (9.6%)	2,867 (9.0%)	<0.001	0.836	0.772-0.904
	No	12,206 (91.9%)	16,799 (90.4%)	29,005 (91.0%)		1	Reference
Diabetes mellitus	Yes	7,104 (53.5%)	6,607 (35.6%)	13,711 (43.0%)	<0.001	2.082	1.989-2.179
	No	6,185 (46.5%)	11,976 (64.4%)	18,161 (57.0%)		1	Reference
Neurological disorder	Yes	1,800 (13.5%)	1,839 (9.9%)	3,639 (11.4%)	<0.001	1.426	1.331-1.529
	No	11,489 (86.5%)	16,744 (90.1%)	28,233 (88.6%)		1	Reference
Chronic respiratory disease	Yes	1,988 (15.0%)	2,140 (11.5%)	4,128 (13.0%)	<0.001	1.352	1.266-1.443
	No	11,301 (85.0%)	16,443 (88.5%)	27,744 (87.0%)		1	Reference
Immunosuppressive disorder	Yes	1,324 (10.0%)	1,595 (8.6%)	2,919 (9.2%)	<0.001	1.179	1.092-1.272
	No	11,965 (90.0%)	16,988 (91.4%)	28,953 (90.8%)		1	Reference
Renal disease	Yes	2,328 (17.5%)	2,359 (12.7%)	4,687 (14.7%)	<0.001	1.461	1.373-1.554
	No	10,961 (82.5%)	16,224 (87.3%)	27,185 (85.3%)		1	Reference
Obesity	Yes	3,353 (25.2%)	3,736 (20.1%)	7,089 (22.2%)	<0.001	1.341	1.272-1.414
	No	9,936 (74.8%)	14,847 (79.9%)	24,783 (77.8%)		1	Reference
Other comorbidities	Yes	7,075 (53.2%)	6,860 (36.9%)	13,935 (43.7%)	<0.001	1.946	1.860-2.036
	No	6,214 (46.8%)	11,723 (63.1%)	17,937 (56.3%)		1	Reference

SARS-CoV-2, severe acute respiratory syndrome 2; OR, odds ratio; 95%CI, 95% confidence interval.
We presented the data as the number of individuals (N) and the percentage (%).
We performed the statistical analysis using the χ^2 test. Alpha=0.05.

Table S15. Association between the case fatality rate due to SARS-CoV-2 infection in hospitalized Brazilian individuals' regarding to the antiviral drugs used to treat the infection and the hospitalization data.

Data	Category	Death	Clinical recovery	Total	*P*	OR	95%CI
Antiviral drugs to treat the infection	Yes	1,854 (14.0%)	3,020 (16.3%)	4,874 (15.3%)	<0.001	0.836	0.785-0.890
	No	11,435 (86.0%)	15,563 (83.7%)	26,998 (84.7%)		1	Reference
Need for intensive care unit	Yes	7,850 (59.1%)	4,256 (22.9%)	12,106 (38.0%)	<0.001	4.859	4.628-5.101
	No	5,439 (40.9%)	14,327 (77.1%)	19,766 (62.0%)		1	Reference
Need for ventilatory support	Invasive	6,239 (46.9%)	1,223 (6.6%)	7,462 (23.4%)	<0.001	20.230	18.610-21.990
	Non-invasive	5,563 (41.9%)	11,463 (61.7%)	17,026 (53.4%)	<0.001	1.925	1.803-2.054
	None required	1,487 (11.2%)	5,897 (31.7%)	7,384 (23.2%)		1	Reference
Discharge criterion	Laboratorial criterion	12,256 (92.2%)	17,492 (94.1%)	29,748 (93.3%)	<0.001	1	Reference
	Clinical criterion	1,033 (7.8%)	1,091 (5.9%)	2,124 (6.7%)		1.351	1.237-1.476

SARS-CoV-2, severe acute respiratory syndrome 2; OR, odds ratio; 95%CI, 95% confidence interval.

We presented the data as the number of individuals (N) and the percentage (%).

We performed the statistical analysis using the χ^2 test. Alpha=0.05.

Association of the outcome and the patients' characteristics with the SARS-CoV-2 infection during the first year of the COVID-19 pandemic in Brazil – Asian individuals.

Table S16. Association between the case fatality rate due to SARS-CoV-2 infection in hospitalized Brazilian individuals' regarding to the demographic data.

Data	Category	Death	Clinical recovery	Total	*P*	OR	95%CI
Sex	Male	1,493 (59.0%)	2,609 (57.0%)	4,102 (57.7%)	0.111	1.083	0.982-1.195
	Female	1,039 (41.0%)	1,967 (43.0%)	3,006 (42.3%)		1	Reference
Age	<1-year-old	5 (0.2%)	14 (0.3%)	19 (0.3%)		1	Reference
	1 to 12 years old	2 (0.1%)	34 (0.7%)	36 (0.5%)	0.082	0.171	0.014-1.193
	13 to 24 years old	9 (0.4%)	126 (2.8%)	135 (1.9%)	0.033	0.203	0.052-0.882
	25 to 60 years old	574 (22.7%)	2,347 (51.3%)	2,921 (41.1%)	0.661	0.685	0.246-1.909
	61 to 72 years old	693 (27.4%)	1,124 (24.6%)	1,817 (25.6%)	0.413	1.726	0.619-4.814
	73 to 85 years old	892 (35.2%)	754 (16.5%)	1,646 (23.2%)	0.028	3.312	1.188-9.238
	+85 years old	357 (14.1%)	177 (3.9%)	534 (7.5%)	<0.001	5.647	2.002-15.930
Place of residence	Urban	2,418 (95.5%)	4,416 (96.5%)	6,834 (96.1%)		1	Reference
	Rural	104 (4.1%)	147 (3.2%)	251 (3.5%)	0.057	1.299	1.000-1.669
	Peri-urban	10 (0.4%)	13 (0.3%)	23 (0.3%)	0.554	1.405	0.615-3.208
Lived in a place with a Flu outbreak	Yes	578 (22.8%)	916 (20.0%)	1,494 (21.0%)	0.005	1.182	1.051-1.330
	No	1,954 (77.2%)	3,660 (80.0%)	5,614 (79.0%)		1	Reference
Nosocomial infection	Yes	60 (2.4%)	55 (1.2%)	115 (1.6%)	<0.001	1.995	1.379-2.886
	No	2,472 (97.6%)	4,521 (98.8%)	6,993 (98.4%)		1	Reference

SARS-CoV-2, severe acute respiratory syndrome 2; OR, odds ratio; 95%CI, 95% confidence interval.

We presented the data as the number of individuals (N) and the percentage (%).

We performed the statistical analysis using the χ^2 test. Alpha=0.05.

Table S17. Association between the case fatality rate due to SARS-CoV-2 infection in hospitalized Brazilian individuals' regarding to the clinical symptoms.

Data	Category	Death	Clinical recovery	Total	*P*	OR	95%CI
Fever	Yes	1,793 (70.8%)	3,358 (73.4%)	5,151 (72.5%)	0.020	0.880	0.790-0.980
	No	739 (29.2%)	1,218 (26.6%)	1,957 (27.5%)		1	Reference
Cough	Yes	1,970 (77.8%)	3,637 (79.5%)	5,607 (78.9%)	0.097	0.905	0.804-1.018
	No	562 (22.2%)	939 (20.5%)	1,501 (21.1%)		1	Reference
Sore throat	Yes	703 (27.8%)	1,501 (32.8%)	2,204 (31.0%)	<0.001	0.787	0.708-0.876
	No	1,829 (72.2%)	3,075 (67.2%)	4,904 (69.0%)		1	Reference
Dyspnea	Yes	2,197 (86.8%)	3,413 (74.6%)	5,610 (78.9%)	<0.001	2.235	1.957-2.552
	No	335 (13.2%)	1,163 (25.4%)	1,498 (21.1%)		1	Reference
Respiratory discomfort	Yes	2,034 (80.3%)	3,020 (66.0%)	5,054 (71.1%)	<0.001	2.104	1.875-2.362
	No	498 (19.7%)	1,556 (34.0%)	2,054 (28.9%)		1	Reference
Oxygen saturation	<95%	2,120 (83.7%)	2,942 (64.3%)	5,062 (71.2%)	<0.001	2.858	2.531-3.227
	≥95%	412 (16.3%)	1,634 (35.7%)	2,046 (28.8%)		1	Reference
Diarrhea	Yes	658 (26.0%)	1,304 (28.5%)	1,962 (27.6%)	0.023	0.881	0.790-0.983
	No	1,874 (74.0%)	3,272 (71.5%)	5,146 (72.4%)		1	Reference
Vomit	Yes	489 (19.3%)	963 (21.0%)	1,452 (20.4%)	0.083	0.898	0.795-1.014
	No	2,043 (80.7%)	3,613 (79.0%)	5,656 (79.6%)		1	Reference
Abdominal pain	Yes	469 (18.5%)	860 (18.8%)	1,329 (18.7%)	0.779	0.982	0.867-1.113
	No	2,063 (81.5%)	3,716 (81.2%)	5,779 (81.3%)		1	Reference
Fatigue	Yes	886 (35.0%)	1,542 (33.7%)	2,428 (34.2%)	0.270	1.059	0.956-1.173
	No	1,646 (65.0%)	3,034 (66.3%)	4,680 (65.8%)		1	Reference
Loss of smell	Yes	554 (21.9%)	1,287 (28.1%)	1,841 (25.9%)	<0.001	0.716	0.639-0.802
	No	1,978 (78.1%)	3,289 (71.9%)	5,267 (74.1%)		1	Reference
Loss of taste	Yes	559 (22.1%)	1,300 (28.4%)	1,859 (26.2%)	<0.001	0.714	0.637-0.800
	No	1,973 (77.9%)	3,276 (71.6%)	5,249 (73.8%)		1	Reference
Other symptoms	Yes	1,069 (42.2%)	2,269 (49.6%)	3,338 (47.0%)	<0.001	0.743	0.674-0.819
	No	1,463 (57.8%)	2,307 (50.4%)	3,770 (53.0%)		1	Reference

SARS-CoV-2, severe acute respiratory syndrome 2; OR, odds ratio; 95%CI, 95% confidence interval.

We presented the data as the number of individuals (N) and the percentage (%).

We performed the statistical data analysis using the χ^2 test. Alpha=0.05.

Table S18. Association between the case fatality rate due to SARS-CoV-2 infection in hospitalized Brazilian individuals' regarding to the comorbidities.

**Data**	**Category**	**Death**	**Clinical recovery**	**Total**	***P***	OR	**95%CI**
Comorbidities (any)	Yes	1,962 (77.5%)	2,766 (60.4%)	4,728 (66.5%)	<0.001	2.252	2.107-2.516
	No	570 (22.5%)	1,810 (39.6%)	2,380 (33.5%)		1	Reference
Cardiopathy	Yes	1,436 (56.7%)	1,868 (40.8%)	3,304 (46.5%)	<0.001	1.899	1.722-2.095
	No	1,096 (43.3%)	2,708 (59.2%)	3,804 (53.5%)		1	Reference
Hematologic disorder	Yes	31 (1.2%)	43 (0.9%)	74 (1.0%)	0.258	1.307	0.821-2.079
	No	2,501 (98.8%)	4,533 (99.1%)	7,034 (99.0%)		1	Reference
Down syndrome	Yes	12 (0.5%)	11 (0.2%)	23 (0.3%)	0.097	1.976	0.871-4.485
	No	2,520 (99.5%)	4,565 (99.8%)	7,085 (99.7%)		1	Reference
Hepatic disorder	Yes	40 (1.6%)	58 (1.3%)	98 (1.4%)	0.280	1.246	0.831-1.870
	No	2,492 (98.4%)	4,518 (98.7%)	7,010 (98.6%)		1	Reference
Asthma	Yes	179 (7.1%)	413 (9.0%)	592 (8.3%)	0.004	0.767	0.639-0.920
	No	2,353 (92.9%)	4,163 (91.0%)	6,516 (91.7%)		1	Reference
Diabetes mellitus	Yes	1,238 (48.9%)	1,554 (4.0%)	2,792 (39.3%)	<0.001	1.861	1.685-2.054
	No	1,294 (51.1%)	3,022 (66.0%0	4,316 (60.7%)		1	Reference
Neurological disorder	Yes	308 (12.2%)	457 (10.0%)	765 (10.8%)	0.005	1.248	1.071-1.455
	No	2,224 (87.8%)	4,119 (90.0%)	6,343 (89.2%)		1	Reference
Chronic respiratory disease	Yes	311 (12.3%)	524 (11.5%)	835 (11.7%)	0.297	1.083	0.932-1.257
	No	2,221 (87.7%)	4,052 (88.5%)	6,273 (88.3%)		1	Reference
Immunosuppressive disorder	Yes	217 (8.6%)	385 (8.4%)	602 (8.5%)	0.820	1.020	0.858-1.214
	No	2,315 (91.4%)	4,191 (91.6%)	6,506 (91.5%)		1	Reference
Renal disease	Yes	336 (13.3%)	543 (11.9%)	879 (12.4%)	0.085	1.136	0.982-1.315
	No	2,196 (86.7%)	4,033 (88.1%)	6,229 (87.6%)		1	Reference
Obesity	Yes	494 (19.5%)	836 (18.3%)	1,330 (18.7%)	0.199	1.068	0.946-1.206
	No	2,038 (80.5%)	3,740 (81.7%)	5,778 (81.3%)		1	Reference
Other comorbidities	Yes	1,315 (51.9%)	1,571 (34.3%)	2,886 (40.6%)	<0.001	2.067	1.872-2.282
	No	1,217 (48.1%)	3,005 (65.7%)	4,222 (59.4%)		1	Reference

SARS-CoV-2, severe acute respiratory syndrome 2; OR, odds ratio; 95%CI, 95% confidence interval.
We presented the data as the number of individuals (N) and the percentage (%).
We performed the statistical analysis using the χ^2 test. Alpha=0.05.

Table S19. Association between the case fatality rate due to SARS-CoV-2 infection in hospitalized Brazilian individuals' regarding to the antiviral drugs used to treat the infection and the hospitalization data.

**Data**	**Category**	**Death**	**Clinical recovery**	**Total**	***P***	**OR**	**95%CI**
Antiviral drugs to treat the infection	Yes	365 (14.4%)	617 (13.5%)	982 (13.8%)	0.275	1.081	0.940-1.243
	No	2,167 (85.6%)	3,959 (86.5%)	6,126 (86.2%)		1	Reference
Need for intensive care unit	Yes	1,584 (62.6%)	988 (21.6%)	2,572 (36.2%)	<0.001	6.068	5.453-6.753
	No	948 (37.4%)	3,588 (78.4%)	4,536 (63.8%)		1	Reference
Need for ventilatory support	Invasive	1,156 (45.7%)	270 (5.9%)	1,426 (20.1%)	<0.001	25.710	21.290-31.040
	Non-invasive	1,127 (44.5%)	2,811 (61.4%)	3,938 (55.4%)	<0.001	2.407	2.070-2.799
	None required	249 (9.8%)	1,495 (32.7%)	1,744 (24.5%)		1	Reference
Discharge criterion	Laboratorial criterion	2,393 (94.5%)	4,330 (94.6%)	6,723 (94.6%)	0.839	1	Reference
	Clinical criterion	139 (5.5%)	246 (5.4%)	385 (5.4%)		1.022	0.826-1.266

SARS-CoV-2, severe acute respiratory syndrome 2; OR, odds ratio; 95%CI, 95% confidence interval.

We presented the data as the number of individuals (N) and the percentage (%).

We performed the statistical analysis using the χ^2 test. Alpha=0.05.

Association of the outcome and the patients' characteristics with the SARS-CoV-2 infection during the first year of the COVID-19 pandemic in Brazil – multiracial backgrounds individuals.

Table S20. Association between the case fatality rate due to SARS-CoV-2 infection in hospitalized Brazilian individuals' regarding to the demographic data.

Data	Category	Death	Clinical recovery	Total	*P*	OR	95%CI
Sex	Male	52,842 (57.4%)	79,008 (55.2%)	131,850 (56.1%)	<0.001	1.093	1.075-1.111
	Female	39,207 (42.6%)	64,051 (44.8%)	103,258 (43.9%)		1	Reference
Age	<1-year-old	206 (0.2%)	1,400 (1.0%)	1,606 (0.7%)		1	Reference
	1 to 12 years old	165 (0.2%)	3,069 (2.1%)	3,234 (1.4%)	<0.001	0.365	0.295-0.453
	13 to 24 years old	698 (0.8%)	4,760 (3.3%)	5,458 (2.3%)	0.998	0.997	0.844-1.177
	25 to 60 years old	27,4421 (29.8%)	81,042 (56.6%)	108,484 (46.1%)	<0.001	2.301	1.987-2.665
	61 to 72 years old	27,379 (29.7%)	30,715 (21.5%)	58,094 (24.7%)	<0.001	6.058	5.229-7.018
	73 to 85 years old	26904 (29.2%)	17981 (12.6%)	44,885 (19.1%)	<0.001	10.170	8.774-11.780
	+85 years old	9,255 (10.1%)	4,092 (2.9%)	13,347 (5.7%)	<0.001	15.370	13.220-17.870
Place of residence	Urban	85,743 (93.1%)	135,253 (94.5%)	220,996 (94.0%)		1	Reference
	Rural	5,935 (6.4%)	7,379 (5.2%)	13,314 (5.7%)	<0.001	1.269	1.225-1.314
	Peri-urban	371 (0.4%)	427 (0.3%)	798 (0.3%)	<0.001	1.371	1.192-1.575
Lived in a place with a Flu outbreak	Yes	24,546 (26.7%)	35,202 (24.6%)	59,748 (25.4%)	<0.001	1.114	1.093-1.135
	No	6,7503 (73.3%)	107,857 (75.4%)	175,360 (74.6%)		1	Reference
Nosocomial infection	Yes	2,372 (2.6%)	2,013 (1.4%)	4,385 (1.9%)	<0.001	1.853	1.745-1.968
	No	89,677 (97.4%)	141,046 (98.6%)	230,723 (98.1%)		1	Reference

SARS-CoV-2, severe acute respiratory syndrome 2; OR, odds ratio; 95%CI, 95% confidence interval.
We presented the data as the number of individuals (N) and the percentage (%).
We performed the statistical analysis using the χ^2 test. Alpha=0.05.

Table S21. Association between the case fatality rate due to SARS-CoV-2 infection in hospitalized Brazilian individuals' regarding to the clinical symptoms.

Data	Category	Death	Clinical recovery	Total	*P*	OR	95%CI
Fever	Yes	66,158 (71.9%)	105,224 (73.6%)	171,382 (72.9%)	<0.001	0.919	0.902-0.936
	No	25,891 (28.1%)	37,835 (26.4%)	63,726 (27.1%)		1	Reference
Cough	Yes	72,360 (78.6%)	116,273 (81.3%)	188,633 (80.2%)	<0.001	0.847	0.829-0.864
	No	19,689 (21.4%)	26,786 (18.7%)	46,475 (19.8%)		1	Reference
Sore throat	Yes	26,729 (29.0%)	46,347 (32.4%)	73,076 (31.1%)	<0.001	0.854	0.839-0.869
	No	65,320 (71.0%)	96,712 (67.6%)	162,032 (68.9%)		1	Reference
Dyspnea	Yes	81,176 (88.2%)	110,180 (77.0%)	191,356 (81.4%)	<0.001	2.228	2.176-2.281
	No	10,873 (11.8%)	32,879 (23.0%)	43,752 (18.6%)		1	Reference
Respiratory discomfort	Yes	75,257 (81.8%)	98,129 (68.6%)	173,386 (73.7%)	<0.001	2.052	2.011-2.094
	No	16,792 (18.2%)	44,930 (31.4%)	61,722 (26.3%)		1	Reference
Oxygen saturation	<95%	76,627 (83.2%)	90,967 (63.6%)	167,594 (71.3%)	<0.001	2.845	2.788-2.904
	≥95%	15,422 (16.8%)	52,092 (36.4%)	67,514 (28.7%)		1	Reference
Diarrhea	Yes	21,882 (23.8%)	37,197 (26.0%)	59,079 (25.1%)	<0.001	0.888	0.871-0.905
	No	70,167 (76.2%)	105,862 (74.0%)	176,029 (74.9%)		1	Reference
Vomit	Yes	17,021 (18.5%)	28,792 (20.1%)	45,813 (19.5%)	<0.001	0.900	0.882-0.920
	No	75,028 (81.5%)	114,267 (79.9%)	189,295 (80.5%)		1	Reference
Abdominal pain	Yes	15,748 (17.1%)	24,209 (16.9%)	39,957 (17.0%)	0.242	1.013	0.991-1.036
	No	76,301 (82.9%)	118,850 (83.1%)	195,151 (83.0%)		1	Reference
Fatigue	Yes	31,478 (34.2%)	44,796 (31.3%)	76,274 (32.4%)	<0.001	1.140	1.120-1.160
	No	60,571 (65.8%)	98,263 (68.7%)	158,834 (67.6%)		1	Reference
Loss of smell	Yes	20,119 (21.9%)	36,873 (25.8%)	56,992 (24.2%)	<0.001	0.806	0.790-0.821
	No	71,930 (78.1%)	106,186 (74.2%)	178,116 (75.8%)		1	Reference
Loss of taste	Yes	20,087 (21.8%)	37,117 (25.9%)	57,204 (24.3%)	<0.001	0.797	0.781-0.813
	No	71,962 (78.2%)	105,942 (74.1%)	177,904 (75.7%)		1	Reference
Other symptoms	Yes	36,569 (39.7%)	66,161 (46.2%)	102,730 (43.7%)	<0.001	0.766	0.753-0.779
	No	55,480 (60.3%)	76,898 (53.8%)	132,378 (56.3%)		1	Reference

SARS-CoV-2, severe acute respiratory syndrome 2; OR, odds ratio; 95%CI, 95% confidence interval.
We presented the data as the number of individuals (N) and the percentage (%).
We performed the statistical data analysis using the χ^2 test. Alpha=0.05.

Table S22. Association between the case fatality rate due to SARS-CoV-2 infection in hospitalized Brazilian individuals' regarding to the comorbidities.

**Data**	**Category**	**Death**	**Clinical recovery**	**Total**	***P***	OR	**95%CI**
Comorbidities (any)	Yes	68,021 (73.9%)	81,052 (56.7%)	149,073 (63.4%)	<0.001	2.166	2.127-2.205
	No	24,028 (26.1%)	62,007 (43.3%)	86,035 (36.6%)		1	Reference
Cardiopathy	Yes	47,909 (52.0%)	50,615 (35.4%)	98,524 (41.9%)	<0.001	1.982	1.949-2.106
	No	44,140 (48.0%)	92,444 (64.6%)	136,584 (58.1%)		1	Reference
Hematologic disorder	Yes	1,045 (1.1%)	1,182 (0.8%)	2,227 (0.9%)	<0.001	1.378	1.268-1.499
	No	91,004 (98.9%)	141,877 (99.2%)	232,881 (99.1%)		1	Reference
Down syndrome	Yes	319 (0.3%)	331 (0.2%)	650 (0.3%)	<0.001	1.500	1.286-1.749
	No	91,730 (99.7%)	142,728 (99.8%)	234,458 (99.7%)		1	Reference
Hepatic disorder	Yes	1,521 (1.7%)	1,646 (1.2%)	3,167 (1.3%)	<0.001	1.443	1.346-1.548
	No	90,528 (98.3%)	141,413 (98.8%)	231,941 (98.7%)		1	Reference
Asthma	Yes	5,345 (5.8%)	11,156 (7.8%)	1,6501 (7.0%)	<0.001	0.729	0.705-0.754
	No	86,704 (94.2%)	131,903 (92.2%)	218,607 (93.0%)		1	Reference
Diabetes mellitus	Yes	42,393 (46.1%)	42,743 (29.9%)	85,136 (36.2%)	<0.001	2.004	1.969-2.038
	No	49,656 (53.9%)	100,316 (70.1%)	149,972 (63.8%)		1	Reference
Neurological disorder	Yes	8,987 (9.8%)	11,819 (8.3%)	20,806 (8.8%)	<0.001	1.201	1.167-1.236
	No	83,062 (90.2%)	131,240 (91.7%)	214,302 (91.2%)		1	Reference
Chronic respiratory disease	Yes	9,876 (10.7%)	13,212 (9.2%)	23,088 (9.8%)	<0.001	1.181	1.149-1.214
	No	82,173 (89.3%)	129,847 (90.8%)	212,020 (90.2%)		1	Reference
Immunosuppressive disorder	Yes	6,422 (7.0%)	9,992 (7.0%)	16,414 (7.0%)	0.942	0.999	0.967-1.032
	No	85,627 (93.0%)	133,067 (93.0%)	218,694 (93.0%)		1	Reference
Renal disease	Yes	11,214 (12.2%)	14,134 (9.9%)	25,348 (10.8%)	<0.001	1.265	1.233-1.299
	No	80,835 (87.8%)	128,925 (90.1%)	209,760 (89.2%)		1	Reference
Obesity	Yes	17,722 (19.3%)	22,249 (15.6%)	39,971 (17.0%)	<0.001	1.295	1.267-1.323
	No	74,327 (80.7%)	120,810 (84.4%)	195,137 (83.0%)		1	Reference
Other comorbidities	Yes	44,955 (48.8%)	46,716 (32.7%)	91,671 (39.0%)	<0.001	1.969	1.935-2.002
	No	47,094 (51.2%)	96,343 (67.3%)	143,437 (61.0%)		1	Reference

SARS-CoV-2, severe acute respiratory syndrome 2; OR, odds ratio; 95%CI, 95% confidence interval.

We presented the data as the number of individuals (N) and the percentage (%).

We performed the statistical analysis using the χ^2 test. Alpha=0.05.

Table S23. Association between the case fatality rate due to SARS-CoV-2 infection in hospitalized Brazilian individuals' regarding to the antiviral drugs used to treat the infection and the hospitalization data.

**Data**	**Category**	**Death**	**Clinical recovery**	**Total**	***P***	**OR**	**95%CI**
Antiviral drugs to treat the infection	Yes	11,588 (12.6%)	17,973 (12.6%)	29,561 (12.6%)	0.855	1.002	0.978-1.208
	No	80,461 (87.4%)	125,086 (87.4%)	205,547 (87.4%)		1	Reference
Need for intensive care unit	Yes	53,753 (58.4%)	29,586 (20.7%)	83,339 (35.4%)	<0.001	5.383	5.286-5.483
	No	38,296 (41.6%)	113,473 (79.3%)	151,769 (64.6%)		1	Reference
Need for ventilatory support	Invasive	44,254 (48.1%)	8,513 (6.0%)	52,767 (22.4%)	<0.001	23.030	22.320-23.760
	Non-invasive	36,796 (40.0%)	85,819 (60.0%)	122,615 (52.2%)	<0.001	1.899	1.854-1.946
	None required	10,999 (11.9%)	48,727 (34.1%)	59,726 (25.4%)		1	Reference
Discharge criterion	Laboratorial criterion	83,980 (91.2%)	131,652 (92.0%)	215,632 (91.7%)	<0.001	1	Reference
	Clinical criterion	8,069 (8.8%)	11,407 (8.0%)	19,476 (8.3%)		1.109	1.076-1.142

SARS-CoV-2, severe acute respiratory syndrome 2; OR, odds ratio; 95%CI, 95% confidence interval.
We presented the data as the number of individuals (N) and the percentage (%).
We performed the statistical analysis using the χ^2 test. Alpha=0.05.

Association of the outcome and the patients' characteristics with the SARS-CoV-2 infection during the first year of the COVID-19 pandemic in Brazil – Indigenous individuals.

Table S24. Association between the case fatality rate due to SARS-CoV-2 infection in hospitalized Brazilian individuals' regarding to the demographic data.

**Data**	**Category**	**Death**	**Clinical recovery**	**Total**	***P***	**OR**	**95%CI**
Sex	Male	284 (36.7%)	548 (47.7%)	832 (43.3%)	<0.001	1.573	1.305-1.895
	Female	489 (63.3%)	600 (52.3%)	1,089 (56.7%)		1	Reference
Age	<1-year-old	20 (2.6%)	53 (4.6%)	73 (3.8%)		1	Reference
	1 to 12 years old	12 (1.6%)	81 (7.1%)	93 (4.8%)	0.031	0.393	0.177-0.869
	13 to 24 years old	18 (2.3%)	95 (8.3%)	113 (5.9%)	0.088	0.502	0.244-1.032
	25 to 60 years old	219 (28.3%)	567 (49.4%)	786 (40.9%)	0.960	1.204	0.598-1.752
	61 to 72 years old	193 (25.0%)	179 (15.6%)	372 (19.4%)	<0.001	2.857	1.643-4.968
	73 to 85 years old	221 (28.6%)	131 (11.4%)	352 (18.3%)	<0.001	4.471	2.559-7.810
	+85 years old	90 (11.6%)	42 (3.7%)	132 (6.9%)	<0.001	5.679	3.020-10.680
Place of residence	Urban	375 (48.5%)	582 (50.7%)	957 (49.8%)		1	Reference
	Rural	393 (50.8%)	553 (48.2%)	946 (49.2%)	0.319	1.103	0.918-1.325
	Peri-urban	5 (0.6%)	13 (1.1%)	18 (0.9%)	0.460	0.597	0.211-1.688
Lived in a place with a Flu outbreak	Yes	318 (41.1%)	387 (33.7%)	705 (36.7%)	0.001	1.374	1.138-1.659
	No	455 (58.9%)	761 (66.3%)	1,216 (63.3%)		1	Reference
Nosocomial infection	Yes	12 (1.6%)	14 (1.2%)	26 (1.4%)	0.536	1.277	0.588-2.776
	No	761 (98.4%)	1,134 (98.8%)	1,895 (98.6%)		1	Reference

SARS-CoV-2, severe acute respiratory syndrome 2; OR, odds ratio; 95%CI, 95% confidence interval.
We presented the data as the number of individuals (N) and the percentage (%).
We performed the statistical analysis using the χ^2 test. Alpha=0.05.

Table S25. Association between the case fatality rate due to SARS-CoV-2 infection in hospitalized Brazilian individuals' regarding to the clinical symptoms.

Data	Category	Death	Clinical recovery	Total	*P*	OR	95%CI
Fever	Yes	633 (81.9%)	887 (77.3%)	1,520 (79.1%)	0.014	1.330	1.058-1.673
	No	140 (18.1%)	261 (22.7%)	401 (20.9%)		1	Reference
Cough	Yes	667 (86.3%)	951 (82.8%)	1,618 (84.2%)	0.042	1.303	1.009-1.684
	No	106 (13.7%)	197 (17.2%)	303 (15.8%)		1	Reference
Sore throat	Yes	310 (40.1%)	445 (38.8%)	755 (39.3%)	0.555	1.058	0.878-1.275
	No	463 (59.9%)	703 (61.2%)	1,166 (60.7%)		1	Reference
Dyspnea	Yes	707 (91.5%)	836 (72.8%)	1,543 (80.3%)	<0.001	3.998	3.010-5.310
	No	66 (8.5%)	312 (27.2%)	378 (19.7%)		1	Reference
Respiratory discomfort	Yes	643 (83.2%)	797 (69.4%)	1,440 (75.0%)	<0.001	2.178	1.737-2.732
	No	130 (16.8%)	351 (30.6%)	481 (25.0%)		1	Reference
Oxygen saturation	<95%	662 (85.6%)	663 (57.8%)	1,325 (69.0%)	<0.001	4.363	3.457-5.505
	≥95%	111 (14.4%)	485(42.2%)	596 (31.0%)		1	Reference
Diarrhea	Yes	151 (19.5%)	297 (25.9%)	448 (23.3%)	0.001	0.696	0.557-0.868
	No	622 (80.5%)	851 (74.1%)	1,473 (76.7%)		1	Reference
Vomit	Yes	113 (14.6%)	259 (22.6%)	372 (19.4%)	<0.001	0.588	0.461-0.749
	No	660 (85.4%)	889 (77.4%)	1,549 (80.6%)		1	Reference
Abdominal pain	Yes	135 (17.5%)	215 (18.7%)	350 (18.2%)	0.508	0.918	0.724-1.165
	No	638 (82.5%)	933 (81.3%)	1,571 (81.8%)		1	Reference
Fatigue	Yes	273 (35.3%)	376 (32.8%)	649 (33.8%)	0.244	1.121	0.925-1.359
	No	500 (64.7%)	772 (67.2%)	1,272 (66.2%)		1	Reference
Loss of smell	Yes	161 (20.8%)	287 (25.0%)	448 (23.3%)	0.034	0.789	0.634-0.983
	No	612 (79.2%)	861 (75.0%)	1,473 (76.7%)		1	Reference
Loss of taste	Yes	163 (21.1%)	289 (25.2%)	452 (23.5%)	<0.001	0.794	0.639-0.988
	No	610 (78.9%)	859 (74.8%)	1,469 (76.5%)		1	Reference
Other symptoms	Yes	244 (31.6%)	491 (42.8%)	735 (38.3%)	<0.001	0.617	0.510-0.748
	No	529 (68.4%)	657 (57.2%)	1,186 (61.7%)		1	Reference

SARS-CoV-2, severe acute respiratory syndrome 2; OR, odds ratio; 95%CI, 95% confidence interval.

We presented the data as the number of individuals (N) and the percentage (%).

We performed the statistical data analysis using the χ^2 test. Alpha=0.05.

Table S26. Association between the case fatality rate due to SARS-CoV-2 infection in hospitalized Brazilian individuals' regarding to the comorbidities.

Data	Category	Death	Clinical recovery	Total	*P*	OR	95%CI
Comorbidities (any)	Yes	453 (58.6%)	485 (42.2%)	938 (48.8%)	<0.001	1.935	1.608-2.328
	No	320 (41.4%)	663 (57.8%)	983 (51.2%)		1	Reference
Cardiopathy	Yes	311 (40.2%)	279 (24.3%)	590 (30.7%)	<0.001	2.097	1.722-2.554
	No	462 (59.8%)	869 (75.7%)	1,331 (69.3%)		1	Reference
Hematologic disorder	Yes	7 (0.9%)	5 (0.4%)	12 (0.6%)	0.200	2.089	0.661-6.606
	No	766 (99.1%)	1,143 (99.6%)	1,909 (99.4%)		1	Reference
Down syndrome	Yes	5 (0.6%)	2 (0.2%)	7 (0.4%)	0.125*	3.728	0.608-39.240
	No	768 (99.4%)	1,146 (99.8%)	1,914 (99.6%)		1	Reference
Hepatic disorder	Yes	10 (1.3%)	7 (0.6%)	17 (0.9%)	0.117	2.136	0.810-5.636
	No	763 (98.7%)	1,141 (99.4%)	1,904 (99.1%)		1	Reference
Asthma	Yes	29 (3.8%)	81 (7.1%)	110 (5.7%)	0.002	0.514	0.337-0.793
	No	744 (96.2%)	1,067 (92.9%)	1,811 (94.3%)		1	Reference
Diabetes mellitus	Yes	272 (35.2%)	241 (21.0%)	513 (26.7%)	<0.001	2.043	1.665-2.508
	No	501 (64.8%)	907 (79.0%)	1,408 (73.3%)		1	Reference
Neurological disorder	Yes	35 (4.5%)	85 (7.4%)	120 (6.2%)	0.011	0.593	0.396-0.889
	No	738 (95.5%)	1,063 (92.6%)	1,801 (93.8%)		1	Reference
Chronic respiratory disease	Yes	47 (6.1%)	88 (7.7%)	135 (7.0%)	0.183	0.780	0.541-1.125
	No	726 (93.9%)	1,060 (92.3%)	1,786 (93.0%)		1	Reference
Immunosuppressive disorder	Yes	32 (4.1%)	63 (5.5%)	95 (4.9%)	0.181	0.744	0.481-1.150
	No	741 (95.9%)	1,085 (94.5%)	1,826 (95.1%)		1	Reference
Renal disease	Yes	59 (7.6%)	97 (8.4%)	156 (8.1%)	0.520	0.895	0.639-1.254
	No	714 (92.4%)	1,051 (91.6%)	1,765 (91.9%)		1	Reference
Obesity	Yes	93 (12.0%)	144 (12.5%)	237 (12.3%)	0.738	0.954	0.722-1.260
	No	680 (88.0%)	1,004 (87.5%)	1,684 (87.7%)		1	Reference
Other comorbidities	Yes	287 (37.1%)	290 (25.3%)	577 (30.0%)	<0.001	1.747	1.434-2.129
	No	486 (62.9%)	858 (74.7%)	1,344 (70.0%)		1	Reference

SARS-CoV-2, severe acute respiratory syndrome 2; OR, odds ratio; 95%CI, 95% confidence interval.

We presented the data as the number of individuals (N) and the percentage (%).

We performed the statistical analysis using the χ^2 test (and * the Fisher Exact test). Alpha=0.05.

Table S27. Association between the case fatality rate due to SARS-CoV-2 infection in hospitalized Brazilian individuals' regarding to the antiviral drugs used to treat the infection and the hospitalization data.

**Data**	**Category**	**Death**	**Clinical recovery**	**Total**	***P***	**OR**	**95%CI**
Antiviral drugs to treat the infection	Yes	108 (14.0%)	160 (13.9%)	268 (14.0%)	0.983	1.003	0.771-1.305
	No	665 (86.0%)	988 (86.1%)	1,653 (86.0%)		1	Reference
Need for intensive care unit	Yes	317 (41.0%)	139 (12.1%)	456 (23.7%)	<0.001	5.046	4.017-6.338
	No	456 (59.0%)	1,009 (87.9%)	1,465 (76.3%)		1	Reference
Need for ventilatory support	Invasive	309 (40.0%)	56 (4.9%)	365 (19.0%)	<0.001	22.070	15.600-31.230
	Non-invasive	342 (44.2%)	604 (52.6%)	946 (49.2%)	<0.001	2.265	1.784-2.875
	None required	122 (15.8%)	488 (42.5%)	610 (31.8%)		1	Reference
Discharge criterion	Laboratorial criterion	695 (89.9%)	1,022 (89.0%)	1,717 (89.4%)	0.537	1	Reference
	Clinical criterion	78 (10.1%)	126 (11.0%)	204 (10.6%)		0.910	0.678-1.227

SARS-CoV-2, severe acute respiratory syndrome 2; OR, odds ratio; 95%CI, 95% confidence interval.
We presented the data as the number of individuals (N) and the percentage (%).
We performed the statistical analysis using the χ^2 test. Alpha=0.05.
